# Antibody decay, T cell immunity and breakthrough infections following two SARS-CoV-2 vaccine doses in inflammatory bowel disease patients treated with infliximab and vedolizumab

**DOI:** 10.1038/s41467-022-28517-z

**Published:** 2022-03-16

**Authors:** Simeng Lin, Nicholas A. Kennedy, Aamir Saifuddin, Diana Muñoz Sandoval, Catherine J. Reynolds, Rocio Castro Seoane, Sherine H. Kottoor, Franziska P. Pieper, Kai-Min Lin, David K. Butler, Neil Chanchlani, Rachel Nice, Desmond Chee, Claire Bewshea, Malik Janjua, Timothy J. McDonald, Shaji Sebastian, James L. Alexander, Laura Constable, James C. Lee, Charles D. Murray, Ailsa L. Hart, Peter M. Irving, Gareth-Rhys Jones, Klaartje B. Kok, Christopher A. Lamb, Charlie W. Lees, Daniel M. Altmann, Rosemary J. Boyton, James R. Goodhand, Nick Powell, Tariq Ahmad, CLARITY IBD study, Klaartje B. Kok, Farjhana Bokth, Bessie Cipriano, Caroline Francia, Nosheen Khalid, Hafiza Khatun, Ashley Kingston, Irish Lee, Anouk Lehmann, Kinnari Naik, Elise Pabriaga, Nicolene Plaatjies, Kevin Samuels, Rebecca Saich, Hayley Cousins, Wendy Fraser, Rachel Thomas, Matthew Brown, Benjamin White, Nikolaos Kirkineziadis, Bernadette Tilley, Rafeeq Muhammed, Rehana Bi, Catherine Cotter, Jayne Grove, Kate Hong, Ruth Howman, Monica Mitchell, Sophie Clayton, Sugrah Sultan, Melanie Rooney, Charlotte Cottrill, Salil Singh, Chris Dawe, Robert Hull, Natalie Silva, Jonathan Manning, Lauren Finlayson, Allison Roebuck, Joy Dawson, Sunil Sonwalkar, Naomi Chambers, Matthew Robinson, Andrew Haigh, Lear Matapure, Tim Raine, Varun George, Christina Kapizioni, Konstantina Strongili, Tina Thompson, Mohamed Ahmed, Christos Kontos, Christophe Bourges, Isabella Barbutti, Megan E. Gozzard, Philip Hendy, Rhian Bull, Patricia Costa, Lisa Davey, Hayley Hannington, Kribashnie Nundlall, Catarina Martins, Laura Avanzi, Jaime Carungcong, Sabrina Barr, Richard Appleby, Emma Johnson, Kath Phillis, Rachel Gascoyne, Amanda Crowder, Amanda Whileman, Ian London, Jenny Grounds, Emmeline Martin, Susie Pajak, Jude Price, Kathryn Cawley, Anjan Dhar, Ellen Brown, Amanda Cowton, Kimberley Stamp, Ben Warner, Carmel Stuart, Louise Lacey, Shanika de Silva, Clare Allcock, Philip Harvey, Lesley Jones, Elise Cooke, Johanne Brooks, Pearl Baker, Hannah Beadle, Carina Cruz, Debbie Potter, Joe Collum, Farzana Masters, Aashish Kumar, Samantha Coetzee, Mihaela Peiu, Becky Icke, Meena Raj, Edward Gaynor, Sibongile Chadokufa, Bonita Huggett, Hamza Meghari, Sara El-Khouly, Fevronia Kiparissi, Waffa Girshab, Andrew Claridge, Emily Fowler, Laura McCafferty, Karolina Christodoulides, Angela Clifford, Patrick Dawson, Sailish Honap, Samuel Lim, Raphael Luber, Karina Mahiouz, Susanna Meade, Parizade Raymode, Rebecca Reynolds, Anna Stanton, Sherill Tripoli, Naomi Hare, Senthuran Balachandran, Emma North, Jessica North, Bria Browne, Ella Jameson, Yih Harn Siaw, Lane Manzano, Jonathan Segal, Ibrahim Al-Bakir, Imran Khakoo, Nora Thoua, Katherine Davidson, Jagrul Miah, Lisa Canclini, Alex Hall, Melony Hayes, Sally Myers, Alison Talbot, Jack Turnbull, Emma Whitehead, Katie Stamp, Alison Pattinson, Verghese Mathew, Leanne Sherris, Angela Harvey, Lucy Hicks, Tara-Marie Byrne, Leilani Cabreros, Hannah Downing-Wood, Sophie Hunter, Hemanth Prabhudev, Sharmili Balarajah, Hajir Ibraheim, Melissa Torkizadeh, Jonathan W. Lo, Zhigang Liu, Helen Sutherland, Elva Wilhelmsen, Katherine Mackintosh, Ajay M. Verma, Juliemol Sebastian, Mohammad Farhad Peerally, Parizade Raymode, Anne-marie Guerdette, Alexandra Kent, Lee Meng Choong, Benedetta Pantaloni, Pantelis Ravdas, Babu Vadamalayan, Stephen Foley, Becky Arnold, Cheryl Heeley, Wayne Lovegrove, Donna Sowton, Lynne Allsop, Heidi Gregory, Philip J. Smith, Giovanna Bretland, Sarah King, Martina Lofthouse, Lindsey Rigby, Sreedhar Subramanian, David Tyrer, Kate Martin, Christopher Probert, Nikolaos Kamperidis, Temi Adedoyin, Manisha Baden, Jeannette Brown, Feba Chacko, Michela Cicchetti, Mohammad Aamir Saifuddin, Priya Yesupatham, Rohit Gowda, Maureen Williams, Karen Kemp, Rima Akhand, Glaxy Gray, Anu John, Maya John, Tasnim Mohammed, Diamond Sathe, Natasha Jones, Jennifer Soren, Michael Sprakes, Julie Burton, Patricia Kane, Stephanie Lupton, Jacqueline Bartholomew, George MacFaul, Diane Scaletta, Loria Siamia, Felicity Williams, Chloe Green, Zeljka Ver, Christopher A. Lamb, Mary Doona, Ashleigh Hogg, Lesley Jeffrey, Andrew King, R. Alexander Speight, Jennifer Doyle, Ruth Owen, Craig Mowat, Debbie Rice, Susan MacFarlane, Anne MacLeod, Samera Mohammed, Shona Murray, Anne Elliott, Mary Anne Morris, Louise Coke, Grace Hindle, Eirini Kolokouri, Catherine Wright, Claire Lee, Nicola Ward, Adele Dann, Melanie Lockett, Charlotte Cranfield, Louise Jennings, Ankur Srivastava, Lana Ward, Nouf Jeynes, Poonam Ranga, Praveen Rajasekhar, Lisa Gallagher, Linda Patterson, Jill Ward, Rae Basnett, Judy Murphy, Lauren Parking, Emma Lawson, Stacey Short, David Devadason, Gordon Moran, Neelam Khan, Lauren Tarr, Charmaine Olivia, Jimmy Limdi, Kay Goulden, Asad Javed, Lauren McKenzie, Pradeep Bhandari, Michelle Baker-Moffatt, Joanne Dash, Alison Le Poidevin, Hayley Downe, Lucille Bombeo, Helen Blackman, Alan Wiles, Hannah Bloxham, Jose Dias, Evelyn Nadar, Hollie Curgenven, Jonathan Macdonald, Shona Finan, Faye McMeeken, Misbah Mahmood, Stephanie Shields, John Paul Seenan, Des DeSilva, Susanna Malkakorpi, Rachel Carson, Simon Whiteoak, Kelli Edger-Earley, Luke Vamplew, Sarah Ingram, Sharon Botfield, Fiona Hammonds, Clare James, Tariq Ahmad, Gemma Aspinall, Sarah Hawkins, Suzie Marriott, Clare Redstone, Halina Windak, Ana-Marie Adam, Hannah Mabb, Charles Murray, Cynthia Diaba, Fexy Joseph, Glykeria Pakou, Yvonne Gleeson, James Berrill, Natalie Stroud, Carla Pothecary, Lisa Roche, Keri Turner, Lisa Deering, Lynda Israel, Evelyn Baker, Sean Cutler, Rina Mardania Evans, Maxine Nash, Georgina Mallison, Anna Roynon, John Gordon, Emma Levell, Silvia Zagalo, Wendy Fraser, Ina Hoad, Nikolaos Kirkineziadis, Richard Russell, Paul Henderson, Margaret Millar, Andrew Fagbemi, Felicia Jennings, Imelda Mayor, Jill Wilson, Christopher Alexakis, Natalia Michalak, John Saunders, Helen Burton, Vanessa Cambridge, Tonia Clark, Charlotte Ekblad, Sarah Hierons, Joyce Katebe, Emma Saunsbury, Rachel Perry, Matthew Brookes, Kathryn Davies, Marie Green, Ann Plumbe, Clare Ormerod, Helen Christensen, Anne Keen, Jonathan Ogor, Alpha Anthony, Emily Newitt, Fiona Trim, Ruth Casey, Katherine Seymour, Edward Fogden, Kalisha Russell, Anne Phillips, Muaad Abdulla, Jeff Butterworth, Colene Adams, Elizabeth Buckingham, Danielle Childs, Alison Magness, Jo Stickley, Nichola Motherwell, Louise Tonks, Hannah Gibson, S. Pajak, Caradog Thomas, Elaine Brinkworth, Lynda Connor, Amanda Cook, Tabitha Rees, Rachel Harford, Emma Wesley, Alison Moss, Jacob Lucas, Claire Lorimer, Maria Oleary, Maxine Dixon, Arvind Ramadas, Julie Tregonning, Olaku Okeke, Wendy Jackson, Ioannis Koumoutsos, Viji George, Swapna Kunhunny, Sophie Laverick, Isla Anderson, Sophie Smith, Kamal Patel, Mariam Ali, Hilda Mhandu, Aleem Rana, Katherine Spears, Joana Teixeira, Richard Pollok, Mark Mencias, Abigail Seaward, Jessica Sousa, Nooria Said, Mark Soomaroo, Valentina Raspa, Asha Tacouri, Nicholas Reps, Rebecca Martin, Christian Selinger, Jenelyn Carbonell, Felicia Onovira, Doris Quartey, Alice L’Anson, Andrew Ashworth, Jessica Bailey, Angie Dunn, Zahid Mahmood, Racheal Campbell, Liane Marsh, Monira Rahman, Sarah Davies, Ruth Habibi, Ellen Jessup-Dunton, Teishel Joefield, Reina Layug, Vinod Patel, Joanne Vere, Victoria Turner, Susan Kilroy, Gareth Walker, Stacey Atkins, Jasmine Growdon, Charlotte McNeill, Rachel Cooney, Lillie Bennett, Louise Bowlas, Sharafaath Shariff, Aileen Fraser, Dwayne Punnette, Charlotte Bishop-Hurman, Elizabeth Undrell, Katherine Belfield, Said Din, Catherine Addleton, Marie Appleby, Johanna Brown, Kathleen Holding, Patricia Hooper, John deCaestecker, Olivia Watchorn, Chris Hayward, Susan Inniss, Lucy Pritchard, Karen Rudge, Amanda Carney, Jervoise Andreyev, Caroline Hayhurst, Carol Lockwood, Lynn Osborne, Amanda Roper, Karen Warner, Julia Hindle, Caroline Watt, Kinga Szymiczek, Shameer Mehta, James Bell, William Blad, Lisa Whitley, Durai Dhamaraj, Mark Baker, Elizabeth John Sivamurugan, Mim Evans, Fraser Cummings, Clare Harris, Amy Jones, Liga Krauze, Sohail Rahmany, Michelle Earl, Jenny Vowles, Audrey Torokwa, Mirela Petrova, Andrew Procter, Jo Stanley, Claudia Silvamoniz, Marion Bettey, Amar Wahid, Zoe Morrison, Rhian Thomas-Turner, Louise Yendle, Jennifer Muller, Marcus Mitchell, John Kirkwood, Anna Barnes, Rakesh Chaudhary, Melanie Claridge, Chiara Ellis, Cheryl Kemp, Ogwa Tobi, Jentus Milton, Emma Johnston, Metod Oblak, Jo Godden, Charlie Lees, Debbie Alexander, Kate Covil, Lauranne Derikx, Sryros Siakavellas, Helen Baxter, Scott Robertson, Linda Smith, Beena Poulose, Anne Colemam, Margareta Balint, Gareth Rhys-Jones, Kerrie Johns, Rachel Hughes, Janet Phipps, Abigail Taylor, Catherine MacPhee, Suzanne Brooks, Katie Smith, Linda Howard, Dianne Wood, Ajay Muddu, Laura Barman, Janine Mallinson, Tania Neale, Diana Ionita, Kerry Elliot, Alison Turnball, Iola Thomas, Kelly Andrews, Jonathon Sutton, Caroline Mulvaney Jones, Julia Roberts, Jeannie Bishop

**Affiliations:** 1 Department of Gastroenterology, Royal Devon and Exeter NHS Foundation Trust, Exeter, UK; 2 Exeter Inflammatory Bowel Disease and Pharmacogenetics Research Group, University of Exeter, Exeter, UK; 3 Department of Gastroenterology, St Marks Hospital and Academic Institute, London, UK; 4 Department of Metabolism, Digestion and Reproduction, Imperial College London, London, UK; 5 Department of Infectious Disease, Imperial College London, London, UK; 6 Department of Immunology and Inflammation, Imperial College London, London, United Kingdom; 7 Department of Biochemistry, Exeter Clinical Laboratory International, Royal Devon and Exeter NHS Foundation Trust, Exeter, UK; 8 IBD Unit, Department of Gastroenterology, Hull University Teaching Hospitals NHS Trust, Hull, UK; 9 Hull York Medical School, University of Hull, Hull, UK; 10 Department of Gastroenterology, Imperial College Healthcare NHS Trust, London, UK; 11 Department of Gastroenterology, Royal Free London NHS Foundation Trust, London, UK; 12 Genetic Mechanisms of Disease Laboratory, The Francis Crick Institute, London, UK; 13 Cambridge Institute of Therapeutic Immunology and Infectious Disease, Jeffrey Cheah Biomedical Centre, Cambridge Biomedical Campus, University of Cambridge, Cambridge, UK; 14 Department of Gastroenterology, Guy’s and St Thomas’ NHS Foundation Trust, London, UK; 15 School of Immunology & Microbial Sciences, King’s College London, London, UK; 16 Department of Gastroenterology, Western General Hospital, NHS Lothian, Edinburgh, UK; 17 Centre for Inflammation Research, The Queen’s Medical Research Institute, The University of Edinburgh, Edinburgh, UK; 18 Department of Gastroenterology, Royal London Hospital, Barts Health NHS Trust, London, UK; 19 Centre for Immunobiology, Blizard Institute, Barts and the London School of Medicine, Queen Mary University of London, London, UK; 20 Department of Gastroenterology, Newcastle upon Tyne Hospitals NHS Foundation Trust, Newcastle upon Tyne, UK; 21 Translational & Clinical Research Institute, Faculty of Medical Sciences, Newcastle University, Newcastle upon Tyne, UK; 22 Institute of Genetic and Molecular Medicine, University of Edinburgh, Edinburgh, UK; 23 Lung Division, Royal Brompton Hospital and Harefield Hospitals, London, UK; 24 Barts Health NHS Trust, London, UK; 25 Barts Health NHS Trust (Paediatric), London, UK; 26 Darlington Memorial Hospital, County Durham, UK; 27 Basingstoke and North Hampshire Hospital, Basingstoke, UK; 28 Birmingham Women’s and Children’s NHS Foundation Trust, Birmingham, UK; 29 Bolton NHS Foundation Trust, Bolton, UK; 30 Borders General Hospital, Scotland, UK; 31 Calderdale and Huddersfield NHS Foundation Trust, Huddersfield, UK; 32 Cambridge University Hospitals NHS Foundation Trust, Cambridge, UK; 33 Chelsea and Westminster Hospital NHS Foundation Trust, London, UK; 34 Chesterfield Royal Hospital, Chesterfield, UK; 35 Countess of Chester Hospital NHS Foundation Trust, Chester, UK; 36 Dartford and Gravesham NHS Trust, Kent, UK; 37 The Dudley Group NHS Foundation Trust, West Midlands, UK; 38 East and North Hertfordshire NHS Trust, Stevenage, UK; 39 East Lancashire Hospitals NHS Trust, Blackburn, UK; 40 Glangwili Hospital, Carmarthen, UK; 41 Great Ormond Street Hospital, London, UK; 42 Great Western Hospitals NHS Foundation Trust, Swindon, UK; 43 Guy’s and St Thomas’ NHS Foundation Trust, London, UK; 44 The Hillingdon Hospitals NHS Foundation Trust, Uxbridge, UK; 45 Homerton University Hospital Foundation Trust, London, UK; 46 Hull University Teaching Hospitals NHS Trust, Hull, UK; 47 Imperial College Healthcare NHS Trust, London, UK; 48 Faculty of Medicine, Imperial College London, London, UK; 49 James Paget University Hospitals NHS Foundation Trust, Great Yarmouth, UK; 50 Kettering General Hospital, Kettering, UK; 51 King’s College Hospital NHS Foundation Trust, London, UK; 52 King’s College Hospital NHS Foundation Trust (Paediatric), London, UK; 53 King’s Mill Hospital, Nottinghamshire, UK; 54 Liverpool University Hospitals NHS Foundation Trust, Liverpool, UK; 55 London North West University Healthcare NHS Trust, London, UK; 56 Maidstone and Tunbridge Wells NHS Trust, Tunbridge Wells, UK; 57 Manchester University NHS Foundation Trust, Manchester, UK; 58 The Mid Yorkshire Hospitals NHS Trust, Wakefield, UK; 59 Milton Keynes University Hospital, Milton Keynes, UK; 60 Newcastle Hospitals NHS Foundation Trust, Newcastle, UK; 61 Ninewells Hospital & Medical School, Dundee, UK; 62 Norfolk and Norwich University Hospitals NHS Foundation Trust, Norwich, UK; 63 North Bristol NHS Trust, Bristol, UK; 64 North Tyneside General Hospital, North Shields, UK; 65 Nottingham University Hospitals NHS Trust, Nottingham, UK; 66 The Pennine Acute Hospitals NHS Trust, Manchester, UK; 67 Portsmouth Hospitals NHS Trust, Portsmouth, UK; 68 The Queen Elizabeth Hospital Kings Lynn NHS Trust, Kings Lynn, UK; 69 Queen Elizabeth University Hospital, Glasgow, UK; 70 Royal Berkshire NHS Foundation Trust, Reading, UK; 71 University Hospitals Dorset NHS Foundation Trust, Dorset, UK; 72 Royal Cornwall Hospitals NHS Trust, Cornwall, UK; 73 Royal Devon and Exeter NHS Foundation Trust, Exeter, UK; 74 Royal Free London NHS Foundation Trust, London, UK; 75 Royal Glamorgan Hospital, Pontyclun, UK; 76 Royal Gwent Hospital, Newport, UK; 77 Royal Hampshire County Hospital, Winchester, UK; 78 Royal Hospital for Sick Children, Edinburgh, UK; 79 Royal Manchester Children’s Hospital, Manchester, UK; 80 Royal Surrey County Hospital, Surrey, UK; 81 Royal United Hospitals Bath, Bath, UK; 82 The Royal Wolverhampton NHS Trust, Wolverhampton, UK; 83 Salford Royal NHS Foundation Trust, Salford, UK; 84 Salisbury District Hospital, Salisbury, UK; 85 Sandwell and West Birmingham NHS Trust, Birmingham, UK; 86 Sheffield Teaching Hospitals NHS Foundation Trust, Sheffield, UK; 87 Shrewsbury and Telford Hospital NHS Trust, Shrewsbury, UK; 88 Singleton Hospital, Swansea, UK; 89 Somerset NHS Foundation Trust, Taunton, UK; 90 South Tees Hospitals NHS Foundation Trust, Middlesbrough, UK; 91 Southend University Hospital NHS Foundation Trust, Essex, UK; 92 St George’s University Hospitals NHS Foundation Trust, London, UK; 93 St George’s University Hospitals NHS Foundation Trust (Paediatric), London, UK; 94 St James’s University Hospital, Leeds, UK; 95 Stockport NHS Foundation Trust, Stockport, UK; 96 Surrey and Sussex Healthcare NHS Trust, Surrey, UK; 97 Tameside and Glossop Integrated Care NHS Foundation Trust, Greater Manchester, UK; 98 Torbay and South Devon NHS Foundation Trust, Torquay, UK; 99 University Hospitals Birmingham NHS Foundation Trust, Birmingham, UK; 100 University Hospitals Bristol NHS Foundation Trust, Bristol, UK; 101 University Hospitals of Derby and Burton NHS Foundation Trust, Derby, UK; 102 University Hospitals of Leicester NHS Trust, Leicester, UK; 103 University Hospitals Plymouth NHS Trust, Plymouth, UK; 104 United Lincolnshire Hospitals NHS Trust, Lincoln, UK; 105 University College London Hospitals NHS Foundation Trust, London, UK; 106 University Hospital Llandough, Penarth, UK; 107 University Hospital Southampton NHS Foundation Trust, Southampton, UK; 108 University Hospital of Wales (Paediatrics), Cardiff, UK; 109 Exeter NIHR Clinical Research Facility, University of Exeter, Exeter, UK; 110 West Hertfordshire Hospitals NHS Trust, Hertfordshire, UK; 111 West Middlesex University Hospital, London, UK; 112 West Suffolk NHS Foundation Trust, Ipswich, UK; 113 Western General Hospital, Edinburgh, UK; 114 Withybush General Hospital, Haverfordwest, UK; 115 Yeovil District Hospital NHS Foundation Trust, Yeovil, UK; 116 York Teaching Hospital NHS Foundation Trust, York, UK; 117 Ysbyty Gwynedd, Bangor, UK

**Keywords:** Vaccines, Inflammatory bowel disease, Humoral immunity, SARS-CoV-2

## Abstract

Anti tumour necrosis factor (anti-TNF) drugs increase the risk of serious respiratory infection and impair protective immunity following pneumococcal and influenza vaccination. Here we report SARS-CoV-2 vaccine-induced immune responses and breakthrough infections in patients with inflammatory bowel disease, who are treated either with the anti-TNF antibody, infliximab, or with vedolizumab targeting a gut-specific anti-integrin that does not impair systemic immunity. Geometric mean [SD] anti-S RBD antibody concentrations are lower and half-lives shorter in patients treated with infliximab than vedolizumab, following two doses of BNT162b2 (566.7 U/mL [6.2] vs 4555.3 U/mL [5.4], p <0.0001; 26.8 days [95% CI 26.2 – 27.5] vs 47.6 days [45.5 – 49.8], p <0.0001); similar results are also observed with ChAdOx1 nCoV-19 vaccination (184.7 U/mL [5.0] vs 784.0 U/mL [3.5], p <0.0001; 35.9 days [34.9 – 36.8] vs 58.0 days [55.0 – 61.3], p value < 0.0001). One fifth of patients fail to mount a T cell response in both treatment groups. Breakthrough SARS-CoV-2 infections are more frequent (5.8% (201/3441) vs 3.9% (66/1682), p = 0.0039) in patients treated with infliximab than vedolizumab, and the risk of breakthrough SARS-CoV-2 infection is predicted by peak anti-S RBD antibody concentration after two vaccine doses. Irrespective of the treatments, higher, more sustained antibody levels are observed in patients with a history of SARS-CoV-2 infection prior to vaccination. Our results thus suggest that adapted vaccination schedules may be required to induce immunity in at-risk, anti-TNF-treated patients.

## Abstract (web summary)

Vaccination is effective in protecting from COVID-19. Here the authors report immune responses and breakthrough infections in twice-vaccinated patients receiving anti-TNF treatments for inflammatory bowel disease, and find dampened vaccine responses that implicate the need of adapted vaccination schedules for these patients.

## Introduction

Vaccination programmes have reduced SARS-CoV-2 transmission, hospitalisation and deaths^1^. Patients treated with immunosuppressive drugs were excluded from the original trials for COVID-19 vaccines^2,3^. Consequently, data relating to the magnitude and durability of immune responses and subsequent vaccine effectiveness in this population are limited^4^.

Drugs targeting tumour necrosis factor (TNF), such as infliximab, are the most frequently prescribed biologic therapies used in the treatment of immune-mediated inflammatory disorders (IMIDs). Observational studies indicate that most patients with inflammatory bowel disease (IBD), an archetypal IMID, mount serological responses following SARS-CoV-2 vaccines, although most were underpowered to discern the impact of specific drugs, including immunomodulators (azathioprine, mercaptopurine and methotrexate) and/or biologic therapies^5–8^. We reported that antibody responses following SARS-CoV-2 infection^9,10^ or a single dose of either the BNT162b2 or ChAdOx1 nCoV-19SARS-CoV-2 vaccines were impaired in anti-TNF treated patients when compared to vedolizumab-treated patients^11^. Vedolizumab, is a gut-selective anti-integrin α4β7 monoclonal antibody that, unlike anti-TNF drugs, is not associated with increased susceptibility to systemic infection or attenuated serological responses to vaccination^12^.

In this work, we show that anti-SARS-CoV-2 spike antibody responses are attenuated and less durable following two doses of the BNT162b2 and ChAdOx1 nCoV-19 SARS-CoV-2 vaccines in infliximab-treated compared with vedolizumab-treated patients with IBD. Irrespective of biologic drug type, one-fifth of all patients do not mount a T cell response and a minority mount neither antibody nor T cell responses. Breakthrough SARS-CoV-2 infections, which are associated with lower antibody levels after the second dose of vaccine, are more common and occur earlier in infliximab-treated patients. Higher and more sustained antibody levels are observed in patients with a history of SARS-CoV-2 infection. Further work to define immunity after third primary and booster vaccine doses is needed to inform the need for adapted vaccination schedules in at-risk anti-TNF treated patients.

## Results

### Patient characteristics

Between September 22, 2020 and December 23, 2020, 7226 patients were recruited to the CLARITY study from 92 UK hospitals^10^. In this analysis we included 2279 infliximab-treated and 1031 vedolizumab-treated participants without a history of prior SARS-CoV-2 infection, who had received uninterrupted biologic therapy since recruitment and had an antibody test between 14 and 70 days after the second dose of either the BNT162b2 and ChAdOx1 nCoV-19 SARS-CoV-2 vaccines. Participant characteristics are shown in Table 1.

**Table 1:** Baseline characteristics of participants who had anti-S receptor-binding domain antibodies measured 2 to 10 weeks following two doses of SARS-CoV-2 vaccine.

Variable	Vedolizumab	Infliximab	*p*
Vaccine			
BNT162b2	40.1% (413/1031)	40.1% (914/2279)	1.0
ChAdOx1 nCoV-19	59.9% (618/1031)	59.9% (1365/2279)	
Age (years)	48.0 (35.2–61.6)	40.2 (30.1–53.1)	<0.0001
Sex			
Female	48.0% (492/1024)	45.9% (1040/2267)	0.15
Male	51.8% (530/1024)	54.1% (1226/2267)	
Intersex	0.0% (0/1024)	0.0% (0/2267)	
Prefer not to say	0.2% (2/1024)	0.0% (1/2267)	
Ethnicity			
White	89.9% (920/1023)	92.5% (2096/2266)	0.037
Asian	6.5% (66/1023)	4.6% (104/2266)	
Mixed	2.4% (25/1023)	1.4% (32/2266)	
Black	0.5% (5/1023)	0.8% (18/2266)	
Other	0.7% (7/1023)	0.7% (16/2266)	
Diagnosis			
Crohn’s disease	36.9% (380/1031)	67.2% (1531/2279)	<0.0001
UC/IBDU	63.1% (651/1031)	32.8% (748/2279)	
Duration of IBD (years)	9.0 (5.0–17.0)	8.0 (3.0–16.0)	0.00017
Age at IBD diagnosis (years)	33.5 (22.8–47.3)	27.6 (20.1–39.6)	<0.0001
Immunomodulators at vaccine	20.4% (210/1031)	57.2% (1304/2279)	<0.0001
5-ASA	33.9% (349/1031)	20.6% (469/2279)	<0.0001
Steroids	5.9% (61/1031)	2.8% (64/2279)	<0.0001
BMI	26.0 (23.0–29.8)	25.9 (22.8–30.0)	0.74
Heart disease	4.9% (50/1023)	2.6% (59/2261)	0.0011
Diabetes	7.6% (78/1023)	3.6% (82/2261)	<0.0001
Lung disease	16.4% (168/1023)	13.1% (296/2261)	0.013
Kidney disease	1.9% (19/1023)	0.8% (17/2261)	0.065
Cancer	1.6% (16/1023)	0.3% (6/2261)	<0.0001
Smoker			
Yes	8.2% (84/1023)	10.8% (245/2261)	0.0010
Not currently	37.7% (386/1023)	30.2% (682/2261)	
Never	54.1% (553/1023)	59.0% (1334/2261)	
Exposure to documented cases of COVID-19	7.8% (80/1023)	8.6% (195/2261)	0.46
Income deprivation score	0.089 (0.055 - 0.146)	0.091 (0.052 - 0.153)	0.92
Active disease (PRO2)	10.4% (102/977)	5.2% (111/2153)	<0.0001
Time between vaccine doses (weeks)	10.9 (9.7–11.1)	11.0 (10.0–11.3)	0.0030
Time from second dose to serum sample (weeks)	5.7 (3.7– 7.7)	5.7 (3.7–7.7)	0.73

Values presented are median (interquartile range) or percentage (numerator/denominator).

*P* values represent the results of a Mann–Whitney *U*, Kruskal–Wallis or Fisher’s exact test.

*UC* ulcerative colitis, *IBDU* IBD unclassified, *IBD* inflammatory bowel disease, *5-ASA* 5-aminosalicylic acid, *BMI* body mass index, *PRO2* IBD disease activity.

Additional analyses are presented for a subset of 211 infliximab-treated and 71 vedolizumab-treated patients included in our T cell experiments (Supplementary Table 1), and a further 530 infliximab-treated and 224 vedolizumab-treated participants who had a history of SARS-CoV-2 infection before vaccination (Supplementary Table 2).

### Anti-SARS-CoV-2-spike (S) antibody level following  two doses of SARS-CoV-2 vaccine

**Fig. 1:**
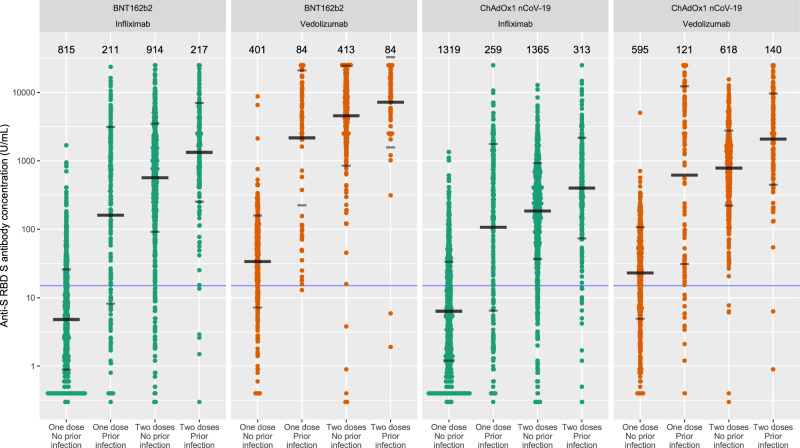
Anti-S RBD antibody concentration stratified by biologic therapy (infliximab vs vedolizumab), type of vaccine, vaccine dose and history of prior SARS-CoV-2 infection. The wider bar represents the geometric mean, while the narrower bars are drawn one geometric standard deviation on either side of the geometric mean. Based on published data using neutralisation assays threshold shown of 15 U/mL was used to determine seroconversion^11^. The biologic treatment infliximab is shown in green and vedolizumab in orange. The number of individuals tested for each group are shown in black at the top of each panel. Source data are provided as a Source Data file.

Overall, the geometric mean [geometric SD] of anti-S receptor-binding domain (RBD) antibody concentration was higher in recipients of two doses of the BNT162b2 than ChAdOx1 nCoV-19 vaccines (1084.1 U/mL [7.6] vs 289.9 U/mL[5.2], *p* < 0.0001). Anti-S RBD antibody concentrations were lower in patients treated with infliximab than in those treated with vedolizumab, following a second dose of BNT162b2 (566.7 U/mL [6.2] vs 4555.3 U/mL [5.4], *p* < 0.0001) and ChAdOx1 nCoV-19 (184.7 U/mL [5.0] vs 784.0 U/mL [3.5], *p* < 0.0001) vaccines (Fig. 1).

Crude sensitivity analyses, excluding patients treated with a concomitant immunomodulator, confirmed lower anti-S RBD antibody concentrations in patients treated with infliximab alone versus vedolizumab alone (BNT162b2 809.1 U/mL [4.9] vs 4691.5 U/mL [5.9], *p* < 0.0001, ChAdOx1 nCoV-19 178.5 U/mL [4.6] vs 778.0 U/mL [3.5], *p* < 0.0001).

After propensity matching for immunomodulator use and the other factors associated with choice of biologic, we confirmed lower anti-S RBD antibody concentrations in infliximab-treated compared to vedolizumab-treated patients (BNT162b2 600.1 U/mL [6.0] vs 4674.1 U/mL [4.7], *p* < 0.0001, ChAdOx1 nCoV-19 195.2 U/mL [4.5] vs 779.2 U/mL [3.6], *p* < 0.0001) (Supplementary Table 3).

**Fig. 2:**
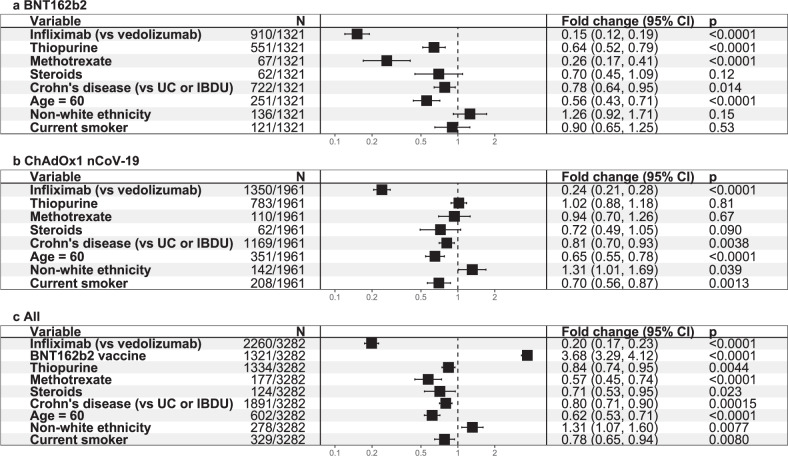
Exponentiated coefficients of linear regression models of log anti-S RBD antibody concentration. Exponentiated coefficients of the linear regression model of log anti-S RBD antibody concentration in participants who received **a** BNT162b2 vaccine. **b** ChAdOx1 nCoV-19 vaccine. **c** either the BNT162b2 or ChAdOx1 nCoV-19 vaccine. The resultant values represent the fold change of antibody concentration associated with each variable (black square). The horizontal solid line through each square represents the 95% confidence interval. Each vaccine was modelled separately, and then a further model was created using all available data. The vertical dotted line represents a fold change of 1. Tests were two-tailed. *p* values were derived from linear regression using the *t*-test statistic and reported without correction for multiple testing. Source data are provided as a Source Data file. UC ulcerative colitis, IBDU IBD unclassified.

Multivariable linear regression analyses in patients without prior SARS-CoV-2 infection confirmed that antibody concentrations were reduced six and four-fold in infliximab-treated compared with vedolizumab-treated participants who received the BNT162b2 (fold change [FC] 0.15 [95% CI 0.12, 0.19], *p* < 0.0001) and ChAdOx1 nCoV-19 ([FC] 0.24 [95% CI 0.21, 0.28], *p* < 0.0001) vaccines (Fig. 2a, b respectively). Age ≥60 years and Crohn’s disease were also independently associated with lower anti-S RBD antibody concentrations in vaccinated participants. Thiopurine or methotrexate use was independently associated with lower anti-S RBD antibody concentrations in participants who received the BNT162b2, but not the ChAdOx1 nCoV-19, vaccine. Current smoking, non-white ethnicity and steroid use were associated with lower anti-S RBD antibody concentrations in participants who received the ChAdOx1 nCoV-19 but not the BNT162b2 vaccine. To assess the effect of vaccine type on antibody responses, we combined our response data in a model that included vaccine type in addition to the significant factors above. Vaccination with the BNT162b2 vaccine compared to the ChAdOx1 nCoV-19 was independently associated with a 3.7 fold [95% CI 3.29–4.12] higher anti-S RBD antibody concentration (*p* < 0.0001) (Fig. 2c).

Seroconversion rates after the first vaccine dose were lower in infliximab-treated compared to vedolizumab-treated participants (Fig. 1). However, administration of a second vaccine dose resulted in a >100-fold and >25-fold increase in antibody concentrations in recipients of the BNT162b2 and ChAdOx1 nCoV-19 vaccines, respectively (Fig. 1). Overall, more infliximab-treated than vedolizumab-treated patients failed to seroconvert after their second vaccine dose (5.9% vs 1.3%, *p* < 0.0001). Seroconversion rates stratified by biologic therapy and vaccine type are reported in Supplementary Table 4.

### Anti-spike T cell responses following two doses of BNT162b2 and ChAdOx1 nCoV-19 SARS-CoV-2 vaccines

**Fig. 3:**
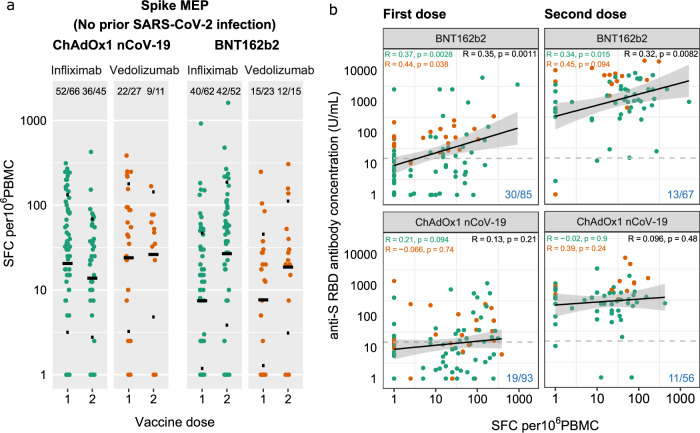
Anti-SARS-CoV-2 spike T cell responses stratified by vaccine platform (BNT162b2 vs ChAdOx1 nCoV-19), biologic therapy (infliximab vs vedolizumab) and vaccine dose (one vs two). **a** Spike MEP T cell responses SFC per 10^6^ PBMC stratified by vaccine platform, biologic therapy (infliximab vs vedolizumab) and the number of vaccine doses. The horizontal bar represents the geometric mean and the narrow bars represent one geometric standard deviation on either side of the geometric mean. The number of T cell responders / total number of individuals tested are shown in black at the top of each panel. **b** Scatterplot demonstrating the correlation between T cell responses against spike MEP pool (SFC per 10^6^ PBMC) and anti-SARS-CoV-2 spike antibody concentration after the first (LHS) and second (RHS) dose of BNT162B2 (top) and ChAdOx1 nCoV-19 (bottom) vaccine. The number of non-T cell responders/total number of individuals tested is shown in blue on the bottom RHS of each panel. The shaded grey band represents the 95% confidence interval. The horizontal dotted line in **b** represents a threshold of 15 U/mL of anti-S1 SARS-CoV-2 antibody. The tests were two-tailed and *p* values were reported without correction for multiple testing. The biologic infliximab is shown in green and vedolizumab is shown in orange. Source data are provided as a Source Data file. MEP mapped epitope peptide, SFC spot forming cells, PBMC peripheral blood mononuclear cell, LHS left-hand side, RHS right-hand side, R Spearman’s rank correlation.

**Fig. 4:**
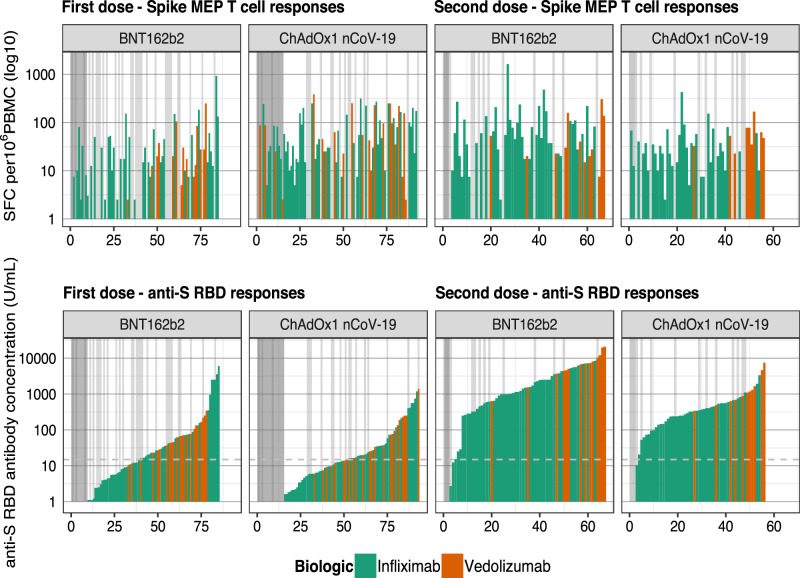
Anti-spike T cell responses ordered by the cumulative magnitude of anti-S RBD following two doses of the BNT162b2 or ChAdOx1 nCoV-19 vaccine show uncoupling of the T cell and antibody responses. Top panel shows T cell responses to spike, and the bottom panel shows anti-S RBD responses plotted for individual study participants ordered by increasing magnitude of anti-S RBD antibody concentration (U/mL). The vertical dark grey bars at the LHS of the panels indicate individuals with no anti-S RBD response. The vertical light grey bars in the panels indicate individuals with no T cell response. The horizontal dotted line represents a threshold shown of 15 U/mL of anti-S RBD. Source data are provided as a Source Data file. LHS left-hand side, MEP mapped epitope peptide, SFC spot forming cells.

There were no significant differences in the magnitude of anti-spike T cell responses observed in infliximab-treated compared with vedolizumab-treated patients after one or two doses of either vaccine (Fig. 3a). The proportion of patients failing to mount detectable T cell responses were similar in both groups (infliximab 19.6% vs. vedolizumab 19.2%). For recipients of one and two doses of the BNT162b2 vaccine, there was a modest positive correlation between T cell responses and antibody concentration. This association was not observed in recipients following either dose of the ChAdOx1 nCoV-19 vaccine (Fig. 3b). When T cell responses were ranked by magnitude of antibody responses, most patients who did not mount an antibody response after the first vaccine dose (indicated by the dark grey bar) had a detectable T cell response (Fig. 4). In addition to the uncoupling of the T cell and antibody responses demonstrated, this analysis emphasised that about one-fifth of participants made no T cell responses irrespective of vaccine used (indicated by the light grey bars). Moreover, a minority of individuals (3/67) 4.5% for BNT162b2 and (1/56) 1.8% for ChAdOx1 nCoV-19 vaccines carry neither detectable antibody nor T cell responses after two doses of vaccine (Figs. 3b, 4).

### Durability of antibody responses following two doses of BNT162b2 and ChAdOx1 nCoV-19 SARS-CoV-2 vaccines

The estimated half-life of anti-S RBD antibodies was shorter in participants receiving the BNT162b2 compared to the ChAdOx1 nCoV-19 vaccines (30.8 days [95% CI 30.3–31.5] vs 40.5 days [95% CI 39.2–41.6], *p* value <0.0001). When stratified by biologic, half-life estimates were shorter in infliximab-treated than vedolizumab-treated patients following two doses of BNT162b2 (26.8 days [95% CI 26.2–27.5] vs 47.6 [95% CI 45.5–49.8], *p* value <0.0001) and ChAdOx1 nCoV-19 (35.9 days [95% CI 34.9–36.8] vs 58.0 days [95% CI 55.0–61.3], *p* value <0.0001) (Supplementary Fig. 1 and Supplementary Table 5).

**Fig. 5:**
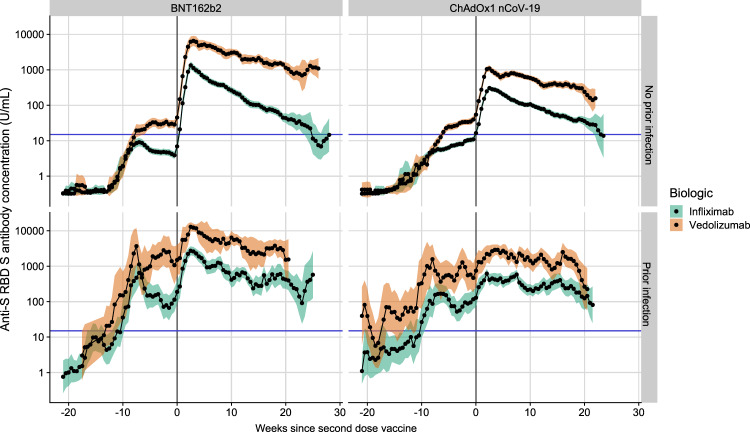
Rolling geometric mean antibody concentration over time from the date of the second dose of the SARS-CoV-2 vaccine (week 0) stratified by biologic therapy (infliximab vs vedolizumab), vaccine and history of prior SARS-CoV-2 infection. Geometric means are calculated using a rolling 15-day window (i.e. 7 days on either side of the day indicated). The shaded areas represent the 95% confidence intervals of the geometric means. The horizontal blue line represents the seroconversion threshold (15 U/mL). The number of participants included at each time point is presented in Supplementary Fig. 2. Overall, data from 4474 participants with no history of prior infection (3029 on infliximab and 1445 on vedolizumab) and 1179 participants with a history of prior infection (833 on infliximab and 346 on vedolizumab) were included in this graph between 22 weeks before and 29 weeks after the second vaccine dose. The biologic treatment infliximab is shown in green and vedolizumab is shown in orange. Source data are provided as a Source Data file.

Overall, following two doses of either vaccine, anti-S RBD antibodies showed minimal decay to the last follow-up in patients treated with vedolizumab (Fig. 5 and Supplementary Fig. 2) and were similar to those observed in participants in the Virus Watch community cohort (Supplementary Fig. 3). However, in infliximab-treated participants, the geometric mean concentrations dropped to the seroconversion threshold by about 25 weeks after the second dose irrespective of the vaccine administered (Fig. 5). Infliximab compared to vedolizumab treatment, current smoking and white ethnicity were associated with a faster fall in anti-S RBD antibody concentration below the seroconversion threshold. (Supplementary Figs. 4, 5).

### Breakthrough SARS-CoV-2 infections following two doses of vaccine

Of 5123 participants without polymerase chain reaction (PCR)-positive or serological evidence of prior SARS-CoV-2 infection, 267 had a first positive SARS-CoV-2 PCR test 2 or more weeks after the second vaccine dose. Overall, 89.2% patients were symptomatic: the most commonly reported symptoms were fatigue (73.7%), anosmia/ageusia (71.4%), fever (57.1%), cough (54.9%), myalgia (45.9%), hoarse voice (30.8%), confusion (27.8%) and chest pains (23.3%). Overall, 1.2% (3/253) of participants with PCR-confirmed infection were hospitalised because of COVID-19.

**Fig. 6:**
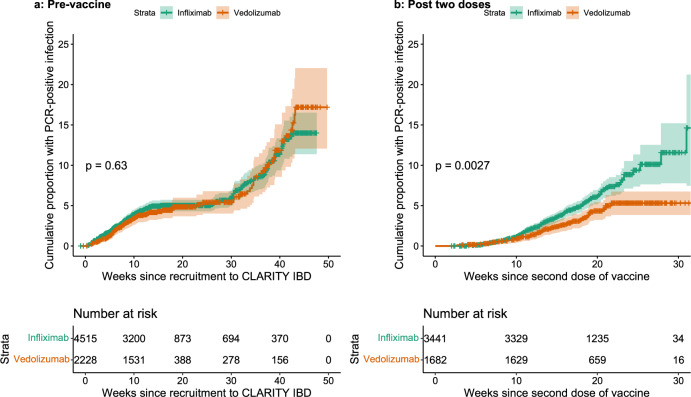
Kaplan–Meier graphs comparing the time to PCR-confirmed SARS-CoV-2 infection stratified by biologic therapy (infliximab vs vedolizumab) in participants before vaccination and after receiving two doses of vaccine. **a** The time to PCR-confirmed SARS-CoV-2 infection in participants who have not received any dose of either vaccine stratified by biologic therapy (infliximab vs vedolizumab). **b** The time to a PCR-confirmed SARS-CoV-2 breakthrough infection in participants following two doses of either vaccine stratified by biologic therapy. The biologic treatment infliximab is shown in green and vedolizumab in orange. The number of participants at each time point are displayed in black at the bottom of each figure. *P* values are calculated using the log-rank test. Source data are provided as a Source Data file.

Breakthrough SARS-CoV-2 infections were more frequent (5.8% (201/3441) vs 3.9% (66/1682), *p* = 0.0039) and the time to breakthrough shorter in patients treated with infliximab than vedolizumab (*p* = 0.0027) (Fig. 6b). In contrast biologic class did not impact on time to PCR-confirmed infection prior to vaccination (*p* = 0.63) (Fig. 6a). In a model that included biologic and vaccine type, shorter time to breakthrough infection was associated with infliximab (Hazard Ratio (HR) 1.52 [95% CI 1.15–2.01], *p* = 0.003) and having received the ChAdOx1 nCoV-19 (HR 1.49 [95% CI 1.15–1.92], *p* = 0.0023) vaccine. Geometric mean [geometric SD] anti-S RBD antibody concentrations measured 2 to 10 weeks after a second vaccine dose were significantly lower in participants who subsequently had a PCR-confirmed breakthrough SARS-CoV-2 infection: for every tenfold rise in anti-S RBD antibody concentration we observed a 0.8-fold reduction in odds of breakthrough infection ([95% CI 0.70–0.99], *p* = 0.03).

### Antibody responses in patients with prior SARS-CoV-2 infection

Amongst patients with a history of SARS-CoV-2 infection before vaccination, geometric mean [SD] anti-S RBD antibody concentrations were lower in infliximab-treated compared with vedolizumab-treated patients after a second dose of BNT162b2 (1330.0 U/mL [5.3] vs 7169.5 U/mL [4.6], *p* < 0.0001) and ChAdOx1 nCoV-19 (399.7 U/mL [5.4] vs 2077.3 [4.6] *p* < 0.0001) vaccines. In all patients, antibody concentrations following vaccination were higher in patients without a history of SARS-CoV-2 infection (Fig. 1). Irrespective of vaccine or biologic type, minimal decay of anti-S RBD antibodies were observed up to a follow-up of 21 weeks.

## Discussion

We have shown that in infliximab-treated patients, anti-SARS-CoV-2 spike antibody responses are attenuated following two doses of the BNT162b2 and ChAdOx1 nCoV-19SARS-CoV-2 vaccines. One-fifth of both infliximab-treated and vedolizumab-treated patients did not mount a T cell response and a small subset of patients had neither antibody nor T cell responses. Antibody half-lives were shorter in infliximab-treated patients. Breakthrough SARS-CoV-2 infections were more common and occurred earlier in infliximab-treated patients who received the ChAdOx1 nCoV-19 vaccine. The risk of breakthrough infection was predicted by lower antibody levels after the second dose of the vaccine. Irrespective of biologic treatment, higher and more sustained antibody levels were observed in patients with a history of SARS-CoV-2 infection.

Sustained antibody responses observed in vaccinated patients with a history of prior SARS-CoV-2 infection indicate that third antigen exposure enhances the serological response. This supports the rationale for prioritising a third dose of vaccine to clinically vulnerable patient populations^13–16^, who otherwise may face further periods of social distancing or hospitalisation following infection. Whilst drawing direct comparisons between IBD patients and patients treated with more potent chemotherapies is limited by the degree to which patients are immunosuppressed, data from solid organ transplant recipients shows that a third dose of vaccine also leads to sustained immune responses^17^.

Irrespective of biologic or immunosuppressant use, and in keeping with the original trials^2,18^, the highest antibody responses were seen in recipients of the BNT162b2 vaccine. Like in the general population, these responses waned more quickly than in the recipients of the ChAdOx1 nCoV-19 vaccine^19^. Unlike the general population^20^, but similar to renal transplant recipients^4^, we did not observe differences in T cell ELISpot responses between recipients of the BNT162b2 and ChAdOx1 nCoV-19 vaccines. The differences observed in breakthrough infection by vaccine type reported here are consistent with the differences in efficacy reported in the respective clinical trials^2,3,21^. The higher peak antibody levels and the lower rate of SARS-CoV-2 breakthrough infections suggest that the BNT162b2 rather than the ChAdOx1 nCoV-19 vaccine should be used for primary vaccination in infliximab-treated patients and, although untested, supports the use of BNT162b2 for third doses in all patients treated with an anti-TNF regardless of the primary vaccine type.

All patients treated with anti-TNF therapy should receive a third primary dose of the SARS-CoV-2 vaccine and our data support recent recommendations that this should occur about 4–8 weeks after the second dose^13,14,16^ during periods of high transmission in the population. Our data demonstrate that patients treated with vedolizumab and infliximab-treated patients with prior SARS-CoV-2 infection have sustained antibody levels beyond 6 months.

When starting a biologic, it would be reasonable to consider differences in SARS-CoV-2 vaccine response as one of the factors when determining which drug to use. For patients who need to start anti-TNF therapy, the benefits of combination immunomodulator therapy should be weighed against the risk of attenuated vaccine response, and whenever feasible, patients should first receive a SARS-CoV-2 vaccine dose. Further research to determine whether timing third vaccine doses towards the end of anti-TNF treatment cycles when drug levels are lowest leads to greater immunogenicity^9^ is needed. Other strategies including the temporary discontinuation of immunomodulators^22^, the use of heterologous vaccines^23^ and adjuvants including the influenza vaccines (ComFluCOV)^24^ need to be studied in immunosuppressed patient groups.

The biology underpinning loss of durable antibody responses and uncoupling of the B cell and T cell responses merit further research. TNF is a pleiotropic cytokine and its activities include maturation of antigen-presenting cells, modulation of T cell responses and stimulation of immunoglobulin synthesis^25–27^. TNF neutralisation, or genetic ablation, results in substantial loss of B-cells in primary follicles in germinal centres, reduced numbers of memory B-cells in the periphery but preserved numbers of T cells^25^. Uncoupling of humoral and T cell immunity to SARS-CoV-2 has been observed in healthy individuals^28^, and although the relative contributions of memory B cell and T cell responses have yet to be fully defined in SARS-CoV-2 immunity, the preservation of T cell immunity reported here should provide some reassurance for anti-TNF treated patients. However, it is noteworthy that one-fifth made no anti-spike T cell response following two doses of either vaccine. Chronic TNF exposure, a feature of many IMIDs, can render T cells anergic and can be reversed by anti-TNF treatment^29^. This may in part explain why the magnitude of T cell responses observed in anti-TNF-treated patients in this study did not differ significantly from patients treated with vedolizumab.

Although our data show major differences in the magnitude and durability of antibody responses, we have not assessed the impact of biologic therapy on specific immunoglobulin classes, antibody neutralisation or mucosal immune responses, which may be impaired, in particular, with anti-a4b7 therapy^30,31^. However, previous studies have demonstrated that anti-RBD antibody levels such as the ones measured in this study, strongly correlate with Wuhan Hu-1 live virus and variant S RBD neutralisation assays^32,33^, and we have demonstrated here that early antibody responses to vaccination correlates with the subsequent risk of breakthrough infection in immunosuppressed patients.

Infliximab was associated with attenuated, less durable vaccine-induced anti-SARS-CoV-2 spike antibody responses and a 50% increase in subsequent breakthrough SARS-CoV-2 infection. Further work to define immunity after third primary and booster vaccine doses is needed to inform the need for adapted vaccination schedules in at-risk anti-TNF treated patients.

## Methods

### Patient and settings

impaCt of bioLogic therApy on saRs-cov-2 Infection and immuniTY (CLARITY) IBD is a UK-wide, multicentre, prospective observational cohort study investigating the impact of infliximab and vedolizumab and/or concomitant immunomodulators (azathioprine, mercaptopurine and methotrexate) on SARS-CoV-2 acquisition, illness and immunity in patients with IBD.

Study methods have been previously described^10,11^. Consecutive patients were recruited at the time of attendance at infusion units between 22 September 2020 and 23 December 2020 (Supplementary Table 1). Patients aged 5 years and over, with a diagnosis of IBD, treated with infliximab or vedolizumab were eligible for inclusion. Follow-up visits coincided with biologic infusions and occurred eight-weekly. Here, we report vaccine-induced antibody responses after the second dose of either the BNT162b2 or ChAdOx1 nCoV-19 vaccines. Participants were eligible for our primary immunogenicity analysis, if they had had an anti-S RBD antibody test between 14 and 70 days after a second-dose vaccine, defined as the second dose of any of the licenced COVID-19 vaccines, 10-14 weeks after the first dose. Anti-S RBD antibody levels were compared with samples from 605 fully vaccinated adult participants from the Virus Watch study, a household community cohort of 10,000 individuals representative of the UK population of England and Wales recruited between 1 June 2020 to 31 August 2021^19^. Peripheral blood mononuclear cells (PBMC) for T cell experiments were collected from patients 4 to 6 weeks after the first and second dose of vaccine at the time of biologic infusions, at selected sites which could facilitate PBMC extraction within 12 h of venepuncture.

### Outcome measures

Our primary outcome was anti-S RBD antibodies 2 to 10 weeks after the second dose of the BNT162b2 or ChAdOx1 nCoV-19 vaccines.

Secondary outcomes were:

(i) the proportion of participants who seroconverted
(ii) anti-spike T cell responses in patients following the first and second dose of vaccines
(iii) the durability of vaccine responses
(iv) risk of breakthrough infections two or more weeks after two doses of vaccine
(v) antibody concentrations and seroconversion rates in patients with PCR or serological evidence of past SARS-CoV-2 infection at, or prior, to the post-vaccination serum sample

### Variables

Variables recorded by participants were demographics (age, sex, ethnicity, comorbidities, height and weight, smoking status, and postcode), IBD disease activity (PRO2), SARS-CoV-2 symptoms aligned to the COVID-19 symptoms study (symptoms, previous testing, and hospital admissions for COVID-19) and vaccine uptake (type and date of primary vaccination). Study sites completed data relating to IBD history (age at diagnosis, disease duration, and phenotype according to the Montreal classifications, previous surgeries, and duration of current biologic and immunomodulator therapy)^10^. We linked our data by NHS number or Community Health Index to Public Health England, Scotland, and Wales archive dates and results of all SARS-CoV-2 PCR tests undertaken and vaccines administered. Data were entered electronically into a purpose-designed REDCap database hosted at the Royal Devon and Exeter NHS Foundation Trust^34^. Participants without access to the internet or electronic device completed their questionnaires on paper case record forms that were subsequently entered by local research teams.

### Laboratory methods

To determine antibody responses specific to vaccination we used the Roche Elecsys Anti-SARS-CoV-2 spike (S) immunoassay^35^ alongside the nucleocapsid (N) immunoassay^36^. This double sandwich electrochemiluminescence immunoassay uses a recombinant protein of the receptor-binding domain on the spike protein as an antigen for the determination of antibodies against SARS-CoV-2. Sample electrochemiluminescence signals are compared to an internal calibration curve and quantitative values are reported as units (U)/mL. In-house assay validation experiments on the Roche Elecsys Anti-SARS-CoV-2 spike (S) immunoassay were performed on 20 samples from healthy individuals who have been vaccinated. This demonstrated:

i. The intra-assay and inter-assay coefficient of variation were 1.3% and 5.6%, respectively
ii. Anti-SARS-CoV-2 (S) antibodies were stable in uncentrifuged blood and serum at ambient temperature for up to seven days permitting postal transport
iii. No effect was observed on recovery of anti-SARS-CoV-2 (S) antibodies following four freeze/thaw cycles
iv. No analytical interference was observed for the detection of anti-SARS-CoV-2 (S) with infliximab or vedolizumab up to 10,000 and 60,000 mg/L, respectively, or with anti-drug antibodies to infliximab or vedolizumab up to 400 and 38 AU/mL, respectively (data not shown).

Seroconversion was defined at a threshold of 15 U/mL. ElecSys Anti-SARS-CoV-2 spike (S) RBD concentrations of greater than or equal to 15 U/ml are associated with neutralisation of ≥20% with a positive predictive value of 99.10% (95% CI: 97.74–99.64)^11^.

At the entry to CLARITY IBD and at follow-up visits, all patients were tested for previous SARS-CoV-2 infection using the Roche Elecsys anti-SARS-CoV-2 (N) immunoassay. We have previously reported that anti-N antibody responses following SARS-CoV-2 natural infection are impaired in patients treated with infliximab or vedolizumab^11^. As such, a threshold 0.12 times above the cut-off index was set, using receiver operator characteristic curve and area under the curve analysis of anti-N antibody results from participants two weeks following a PCR-confirmed infection to maximise specificity, beyond which patients were deemed to have had prior SARS-CoV-2 infection (Supplementary Fig. 6). Patients with a PCR test confirming SARS-CoV-2 infection at any time prior to vaccination were deemed to have evidence of past infection irrespective of any antibody test result. Breakthrough infections were defined by a positive SARS-CoV-2 PCR test 2 or more weeks after the second vaccine dose.

### Peripheral blood mononuclear cell isolation

Whole blood was collected in lithium heparin tubes and PBMCs were isolated by density-gradient centrifugation using Lymphoprep^TM^ (Stem Cell Technologies) layered onto SepMate^TM^ (Stem Cell Technologies) tubes. PBMC isolation was performed within 12 h of venepuncture. Purified PBMCs were cryopreserved in 10% DMSO/50% FBS and stored in liquid nitrogen pending batch analysis.

### Spike-peptide specific T cell responses

IFN-γ T cell ELISpot assays were performed using pre-coated plates (Mabtech 3420-2APT) and using the protocol described previously^28,32^. Two-hundred thousand cells were seeded per well and cells were stimulated with a peptide pool, containing 18 peptides derived from SARS-CoV-2 spike protein^37^ at a concentration of 10 μg/ml/peptide; the peptide pool utilises a mapped epitope pool (MEP) or 12–20mer peptides, mapped as eliciting high-prevalence CD4 responses covering diverse HLA-II haplotypes^28,32^. Use of this spike MEP in otherwise healthy SARS-CoV-2 seropositive individuals elicits a T cell response in 83% of individuals at 16–18 weeks after natural SARS-CoV-2 infection and 91% of healthy individuals 2–3 weeks after two-dose vaccination with seronegative individuals showing a level of response indistinguishable from pre-pandemic controls^28,32^. Plates were cultured for 18–20 h before development and data were collected using an AID classic ELISpot plate reader (Autoimmun Diagnostika GMBH). Results are expressed as differences in (delta) spot forming cells (SFC) per 10^6^ PBMC between peptide stimulation and a media-only control. A response below 2 standard deviations of the media-only control wells was deemed to be a null response. Data were excluded if the response to the positive control anti-CD3 stimulation was <200 SFC per 10^6^ PBMCs.

### Sample size

The sample size for CLARITY IBD was based on the number of participants required to demonstrate a difference in the impact of infliximab and vedolizumab on seroprevalence and seroconversion following SARS-CoV-2 infection, with an estimated background seroprevalence of 0.05. We calculated that a sample of 6970 patients would provide 80% power to detect differences in the seroprevalence of SARS-CoV-2 antibodies in infliximab-treated compared with vedolizumab-treated patients, whilst controlling for immunomodulator status at the 0.05 significance level.

### Statistical analyses

Analyses were undertaken using R 4.1.2 (R Foundation for Statistical Computing, Vienna, Austria). All tests were two-tailed and *p* values were reported without correction for multiple testing. *P* values <0.05 were considered significant. We included patients with missing clinical data in analyses for which they had data and have specified the denominator for each variable. Anti-S RBD antibody concentrations are reported as geometric means and standard deviations. Other continuous data are reported as a median and interquartile range, and discrete data as numbers and percentages, unless otherwise stated.

Univariable analyses, using Spearman’s rank correlation coefficients, and *t*-tests of log-transformed anti-S RBD antibody concentration were used to identify demographic, disease, vaccine and treatment-related factors associated with the concentration of anti-S RBD antibodies across the cohort. Crude sensitivity analyses excluding patients treated without a concomitant immunomodulator were undertaken to control for the effect of immunomodulator use on anti-S RBD antibody concentrations. Propensity matching was used to account for the other significant differences in baseline variables between infliximab-treated and vedolizumab-treated patients using the MatchIt package^38^. A priori, patients were matched exactly on diagnosis, immunomodulator use, and then using optimal matching, on age, the number of comorbidities, ethnicity, and presence of active disease. Multivariable linear regression models were used to identify factors independently associated with log anti-S RBD concentration. A priori, we included age, ethnicity, biologic medication and immunomodulator use. Results are presented after exponentiation so that the coefficients of the model correspond to the fold change (FC) associated with each binary covariate. For age, a cut-off was chosen based on a graphical inspection of the relationship between age and anti-S RBD antibody concentrations.

Mann–Whitney *U-*test was used to compare the magnitude of T cell response (SFC/10^6^ PBMCs) stratified by treatment and vaccine received, and Spearman’s rank correlation coefficient was calculated to determine the correlation between antibody and T cell responses.

Anti-S RBD antibody half-lives were estimated using an exponential model of decay. Linear mixed models were fit using the lme4 and lmerTest package, with biologic treatment and vaccine type as fixed effects and each subject as a random effect. Each of these effects were estimated independently for gradient and intercept. 95% confidence intervals of fixed effects were calculated using likelihood ratios. *P* values for comparison of half-lives were estimated from the full linear mixed-effects model that incorporated vaccine, biologic drug and prior SARS-CoV-2 infection status.

We visualised the durability of antibody responses by calculating 15-day rolling geometric mean anti-S RBD antibody concentrations. For this analysis we included participants who had an antibody test carried out between 1 and 70 days after the second vaccine dose. Cox proportional hazard regression models were used to identify the demographic, disease and treatment-related factors associated with the time to fall in anti-S RBD antibody concentration below the seroconversion threshold.

Kaplan–Meier curves and Cox proportional hazard regression model was used to identify treatment-related factors associated with time to breakthrough infection. A linear regression model of log-transformed geometric mean anti-S RBD antibody concentration was used to determine the risk of breakthrough infections.

Where appropriate the same analyses were used to compare antibody responses in participants with PCR evidence of SARS-CoV-2 infection at any time prior to vaccination.

### Ethical consideration

Patients were included after providing informed, written consent and compensation for participation was not provided. The sponsor was the Royal Devon and Exeter NHS Foundation Trust. The Surrey Borders Research Ethics committee approved the study (REC reference: REC 20/HRA/3114) in September 2020. The protocol is available online at https://www.clarityibd.org. The study was registered with the ISRCTN registry (10.1186/ISRCTN45176516).

### Reporting summary

Further information on research design is available in the Nature Research Reporting Summary linked to this article.

## Supplementary information

Supplementary Information

Reporting Summary

## Data Availability

The study protocol including the statistical analysis plan is available at https://www.clarityibd.org/. Individual participant de-identified data that underlie the results reported in this article will be available immediately after publication for a period of 5 years. Due to the sensitive nature of the data, this will be made available to investigators whose proposed use of the data has been approved by an independent review committee. Analyses will be restricted to the aims in the approved proposal. Proposals should be directed to tariq.ahmad1@nhs.net. To gain access data requestors will need to sign a data access agreement. Data from the Virus Watch study is currently being archived on the Office of National Statistics Secure Research Service and will be available shortly. Source data are provided with this paper in the Source Data file. Source data are provided with this paper.

## Source data

Source Data

## Code availability

Statistical analyses were undertaken in R 4.1.2 (R Foundation for Statistical Computing, Vienna, Austria. Code has been made available at: https://github.com/exeteribd/clarityibd-public.
