# Supplementary material for: Antibody decay, T cell immunity and breakthrough infections following two SARS-CoV-2 vaccine doses in inflammatory bowel disease patients treated with infliximab and vedolizumab
Source: Nat Commun. 2022 Mar 16;13:1379. doi: 10.1038/s41467-022-28517-z (PMC8927425; doi:10.1038/s41467-022-28517-z)
Supplement: Supplementary file 1 — Supplementary Information [file 41467_2022_28517_MOESM1_ESM.pdf]

Supplementary Information for 'Antibody decay, T cell immunity and breakthrough infections following two SARS-CoV-2 vaccine doses in inflammatory bowel disease patients treated with infliximab and vedolizumab'

Simeng Lin[1,2†], Nicholas A Kennedy[1,2†], Aamir Saifuddin[3,4†] Diana Muñoz Sandoval[5†], Catherine J Reynolds[5], Rocio Castro Seoane[6], Sherine H Kottoor[4], Franziska P Pieper[5], Kai-Min Lin[5], David K. Butler[5], Neil Chanchlani[1,2], Rachel Nice[2,7], Desmond Chee[1,2], Claire Bewshea[2], Malik Janjua [1,2], Timothy J McDonald[7], Shaji Sebastian[8,9], James L Alexander[4,10], Laura Constable[4], James C Lee[11,12,13], Charles D Murray[11], Ailsa L Hart[3], Peter M Irving[14,15], Gareth-Rhys Jones[16,17], Klaartje B Kok[18,19], Christopher A Lamb[20,21], Charlie W Lees[16,22], Daniel M Altmann[6], Rosemary J Boyton[5,23†], James R Goodhand[1,2†], Nick Powell[4,10†] , Tariq Ahmad[1,2*†], CLARITY IBD study[#].

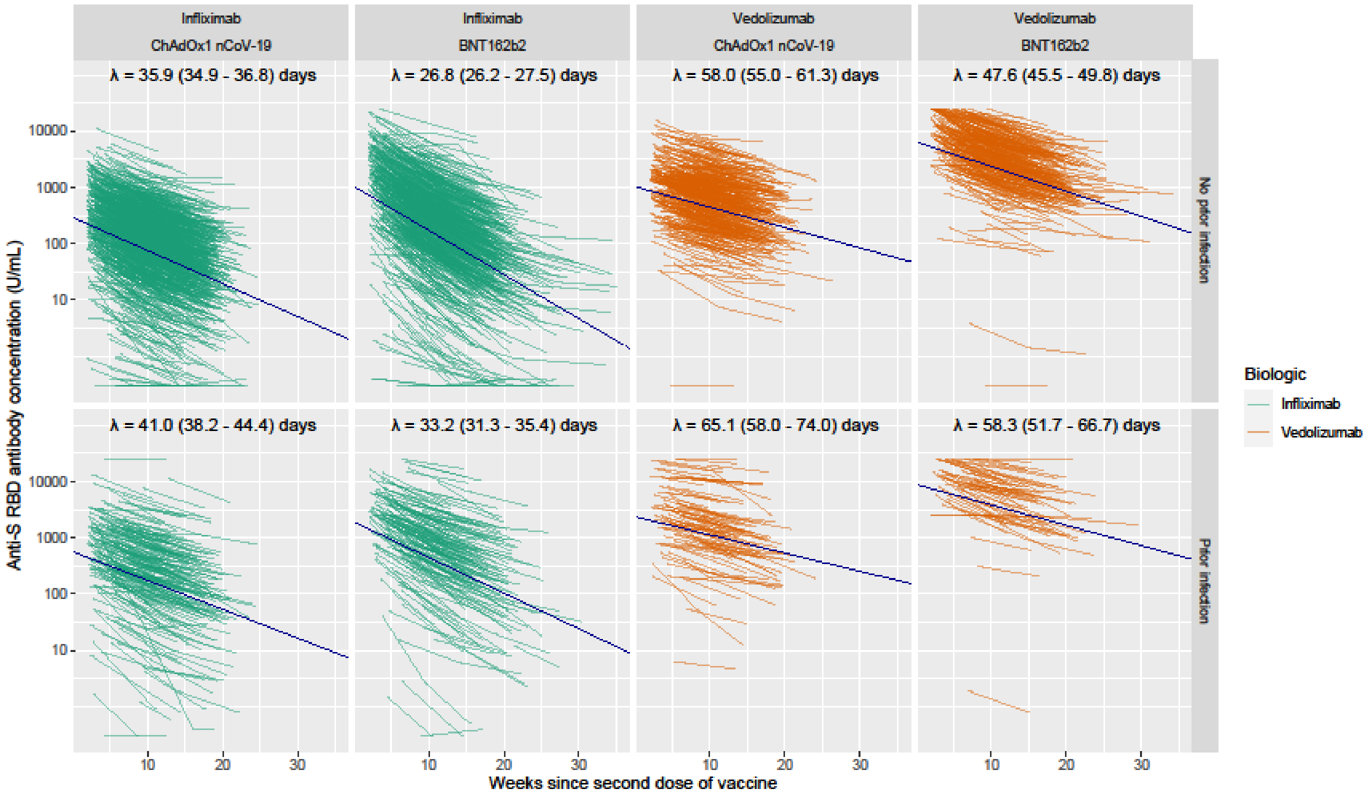

Supplementary Figure 1: Model estimates of the rate of anti-S RBD antibody decay over time, stratified by biologic therapy (infliximab vs vedolizumab), vaccine, and history of prior SARS-CoV-2 infection. The solid blue line represents the model estimates of the rate of anti-S RBD antibody decay over time using a linear mixed effects model with log anti-S RBD antibody as the dependent variable and individual as a random effect. λ represents the half-life calculated from the inverse of the gradient of the model. The biologic treatment infliximab is shown in green and vedolizumab in orange. Source data are provided as a Source Data file.

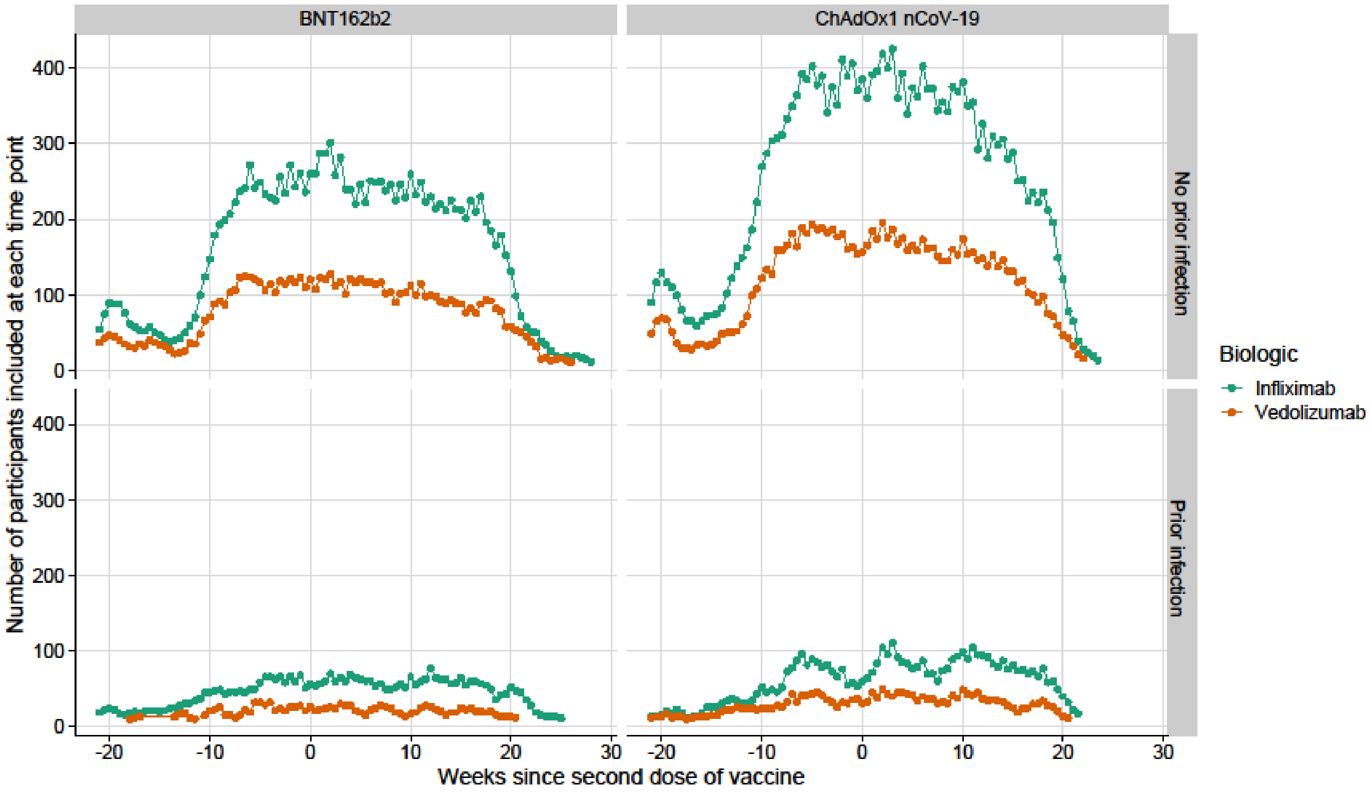

Supplementary Figure 2: Line graph showing the number of participants included at each time point in Figure 5 of the manuscript. The biologic treatment infliximab is shown in green and vedolizumab in orange. Source data are provided as a Source Data file.

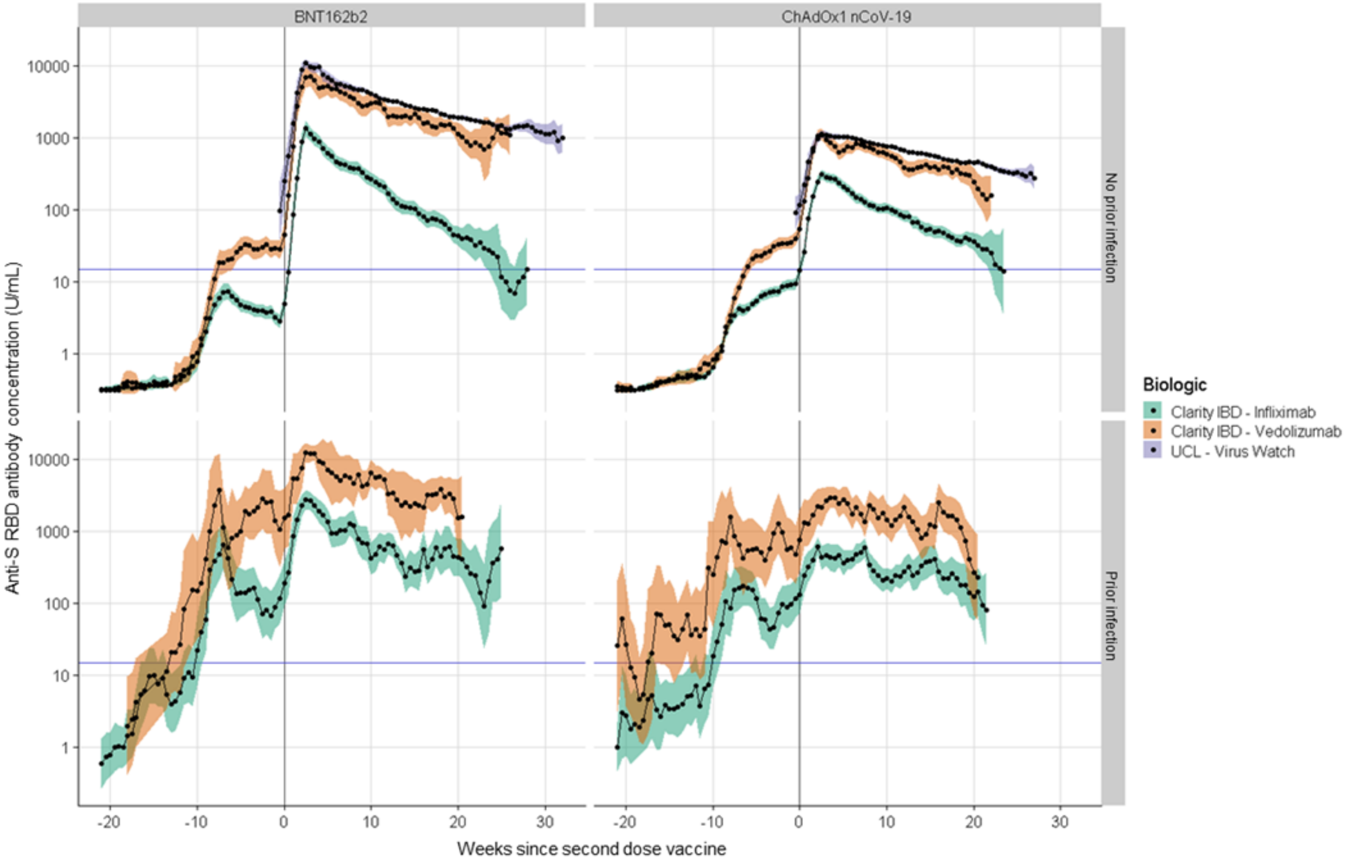

Supplementary Figure 3: Rolling geometric mean antibody concentration over time of participants on biologic therapy (infliximab vs vedolizumab) compared with 605 participants of the Virus Watch community cohort vaccine, timed from second dose SARS-CoV-2 vaccine (week 0), stratified by vaccine and history of prior SARS-CoV-2 infection. Geometric means are calculated using a rolling 15-day window (i.e. 7 days either side of the day indicated). The shaded areas represent the 95% confidence intervals of the geometric means. The horizontal blue line represents the seroconversion threshold (15 U/mL). The biologic treatment infliximab is shown in green and vedolizumab is shown in orange. Participants of the Virus Watch community cohort are shown in purple.

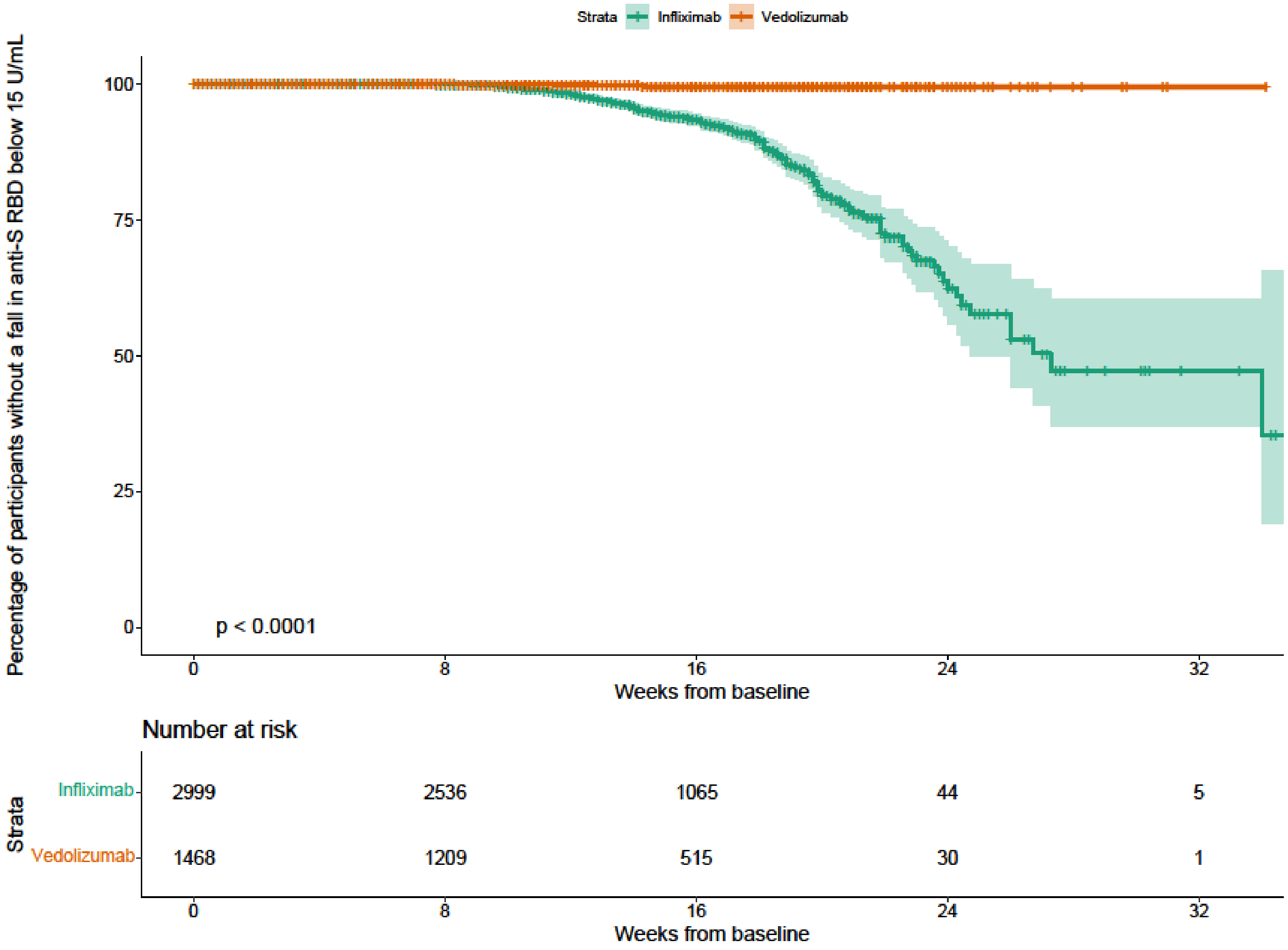

Supplementary Figure 4: Kaplan-Meier graphs showing the time to the anti-S RBD antibody concentration falling below the seroconversion threshold of 15U/mL following the second dose of SARS-CoV-2 vaccine. The shaded areas represent the 95% confidence intervals of the percentage of participants at each time point. The biologic treatment infliximab is shown in green and vedolizumab in orange. The number of participants at each time point are displayed in black at the bottom of each figure. P-values are calculated using log-rank test. Source data are provided as a Source Data file.

Variable	N	HR (95% CI)	p
**Infliximab (vs vedolizumab)**	2979/4433	21.67 (8.85, 53.06)	<0.0001
**Immunomodulator**	2012/4433	1.06 (0.82, 1.38)	0.65
**Crohn's disease (vs UC or IBDU)**	2512/4433	1.24 (0.93, 1.64)	0.14
**Non-white ethnicity**	452/4433	0.40 (0.23, 0.70)	0.0015
**Current smoker**	421/4433	1.86 (1.31, 2.63)	0.00048

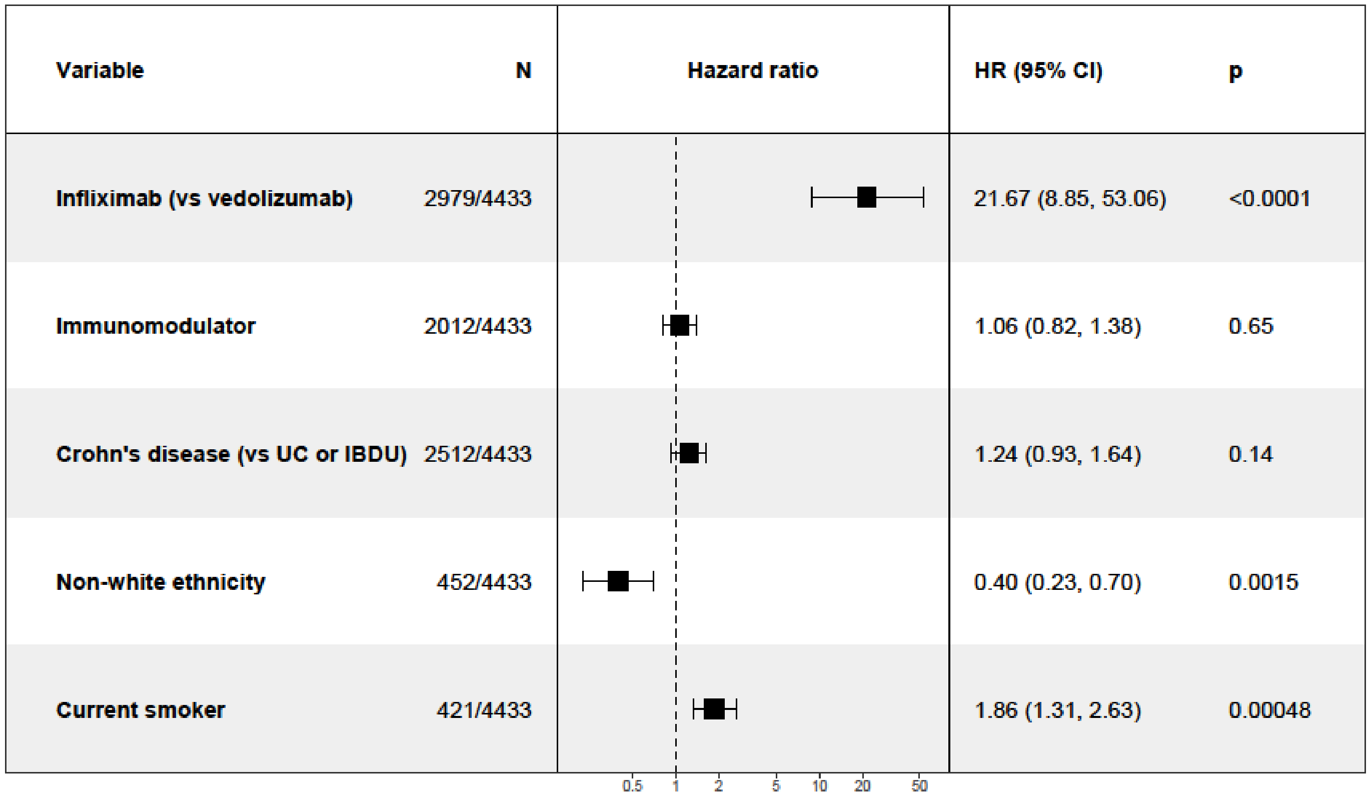

Supplementary Figure 5: Cox proportional hazards model of variables associated with anti-S RBD antibody concentration falling below the seroconversion threshold of 15U/mL after the second dose of SARS-CoV-2 vaccine. The resultant values represent the hazard ratio of anti-S RBD antibody concentration falling below the seroconversion threshold (black square) associated with each variable. The horizontal solid line through each square represents the 95% confidence interval. Tests were two-tailed and p-values calculated using log-rank test without correction for multiple testing. Source data are provided as a Source Data file. Abbreviations: UC = ulcerative colitis; IBDU = inflammatory bowel disease unclassified.

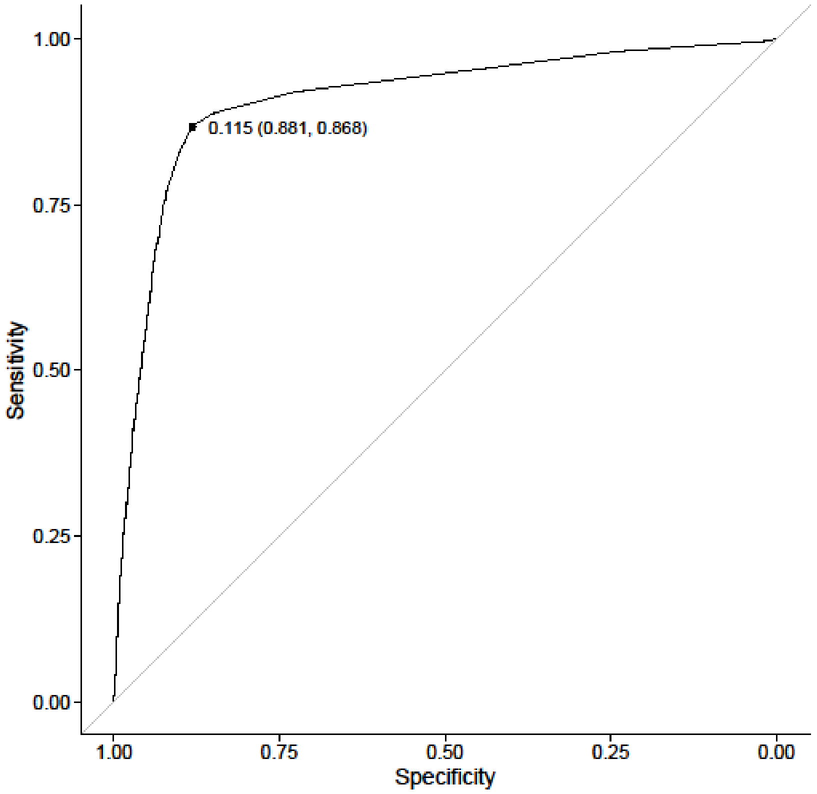

Supplementary Figure 6: Receiver operator characteristic curve of anti-N antibody results from participants two weeks following a PCR-confirmed infection. The light grey line is the reference line. A threshold of 0.12 times the cut-off index provides 100% specificity for determining prior SARS-CoV-2 infection. Source data are provided as a Source Data file.

**Variable**	**Vedolizumab**	**Infliximab**	**p**
Vaccine			
BNT162b2	49.3% (35/71)	49.3% (104/211)	1.0
ChAdOx1 nCoV-19	50.7% (36/71)	50.7% (107/211)	
Age (years)	45.1 (34.0 - 54.5)	38.1 (30.0 - 48.9)	0.0012
Sex			
Female	47.9% (34/71)	39.8% (84/211)	0.27
Male	52.1% (37/71)	60.2% (127/211)	
Intersex	0.0% (0/71)	0.0% (0/211)	
Prefer not to say	0.0% (0/71)	0.0% (0/211)	
Ethnicity			
White	80.3% (57/71)	82.5% (174/211)	0.83
Asian	15.5% (11/71)	11.8% (25/211)	
Mixed	1.4% (1/71)	1.4% (3/211)	
Black	2.8% (2/71)	2.4% (5/211)	
Other	0.0% (0/71)	1.9% (4/211)	
Diagnosis			
Crohn's disease	28.2% (20/71)	67.8% (143/211)	<0.0001
UC/IBDU	71.8% (51/71)	32.2% (68/211)	
Duration of IBD (years)	11.0 (6.5 - 21.0)	10.0 (5.0 - 18.0)	0.12
Age at IBD diagnosis (years)	27.8 (20.2 - 39.4)	24.8 (17.2 - 34.0)	0.023
Immunomodulators at vaccine	10.1% (7/69)	62.6% (132/211)	<0.0001
5-ASA	31.9% (22/69)	19.4% (41/211)	0.045
Steroids	7.2% (5/69)	2.4% (5/211)	0.070
BMI	25.6 (22.0 - 28.9)	24.6 (21.8 - 27.1)	0.38
Heart disease	2.8% (2/71)	1.9% (4/211)	0.64
Diabetes	9.9% (7/71)	3.3% (7/211)	0.051
Lung disease	21.1% (15/71)	13.7% (29/211)	0.18
Kidney disease	4.2% (3/71)	0.9% (2/211)	0.10
Cancer	2.8% (2/71)	0.0% (0/211)	0.063
Smoker			
Yes	4.2% (3/71)	9.0% (19/211)	0.028
Not currently	38.0% (27/71)	22.3% (47/211)	
Never	57.7% (41/71)	68.7% (145/211)	
Exposure to documented cases of COVID-19	8.5% (6/71)	4.3% (9/210)	0.22
Income deprivation score	0.092 (0.048 - 0.163)	0.084 (0.046 - 0.140)	0.26
Active disease (PRO2)	6.2% (4/65)	2.5% (5/201)	0.23
Time between vaccine doses (weeks)	10.9 (10.0 - 11.1)	10.7 (9.9 - 11.2)	0.35

Supplementary Table 1: Baseline characteristics of unique participants included in T cell studies stratified by biologic treatment at time of first vaccine dose

Abbreviations: IBD = inflammatory bowel disease; 5-ASA = 5-aminosalicylic acid; BMI = Body Mass Index; PRO2 = IBD disease activity. Values presented are median (interquartile range) or percentage (numerator/denominator). All tests are two-sided. P values represent the results of a Mann Whitney U, Kruskal Wallis or Fisher's exact test.

Variable	Vedolizumab	Infliximab	p
Vaccine			
BNT162b2	37.5% (84/224)	40.9% (217/530)	0.42
ChAdOx1 nCoV-19	62.5% (140/224)	59.1% (313/530)	
Peak antibody concentration (U/mL)			
Anti-N antibody concentration prior to first dose	0.9 (14.2)	0.3 (5.4)	<0.0001
Anti-S RBD antibody concentration prior to first dose	4.8 (20.9)	1.3 (7.1)	<0.0001
Anti-N antibody concentration prior to second dose	2.7 (16.5)	0.6 (6.4)	<0.0001
Mode of SARS-CoV-2 diagnosis prior to second dose			
Positive PCR test	34.8% (78/224)	30.9% (164/530)	0.31
Anti-N antibody concentration ≥0.12	97.3% (218/224)	95.8% (508/530)	0.40
Positive PCR test and anti-N antibody concentration ≥0.12	34.8% (78/224)	30.9% (164/530)	0.31
Age (years)	43.4 (34.3 - 56.3)	38.8 (29.4 - 51.7)	<0.0001
Sex			
Female	53.1% (119/224)	43.6% (230/528)	0.033
Male	46.9% (105/224)	56.2% (297/528)	
Prefer not to say	0.0% (0/224)	0.0% (0/528)	
Ethnicity			
White	88.8% (198/223)	85.8% (453/528)	0.83
Asian	7.6% (17/223)	8.3% (44/528)	
Mixed	1.3% (3/223)	2.5% (13/528)	
Black	1.8% (4/223)	2.8% (15/528)	
Other	0.4% (1/223)	0.6% (3/528)	
Diagnosis			
Crohn's disease	32.6% (73/224)	66.4% (352/530)	<0.0001
UC/IBDU	67.4% (151/224)	33.6% (178/530)	
Duration of IBD (years)	9.0 (5.0 - 16.0)	8.0 (3.0 - 15.0)	0.028
Age at IBD diagnosis (years)	31.4 (23.1 - 42.8)	27.6 (20.3 – 38.0)	0.0013
Immunomodulators at vaccine	17.4% (39/224)	57.2% (303/530)	<0.0001
5-ASA	35.3% (79/224)	21.9% (116/530)	0.00018
Steroids	5.4% (12/224)	3.2% (17/530)	0.21
BMI	27.1 (24.2 - 31.8)	25.8 (23.4 - 30.1)	0.0078
Heart disease	4.5% (10/222)	2.5% (13/527)	0.16
Diabetes	5.8% (13/223)	3.8% (20/528)	0.24
Lung disease	19.7% (44/223)	10.6% (56/528)	0.0014
Kidney disease	1.8% (4/223)	0.6% (3/528)	0.20
Cancer	1.3% (3/223)	0.4% (2/528)	0.16
Smoker			
Yes	7.6% (17/223)	11.0% (58/528)	0.14
Not currently	35.0% (78/223)	28.8% (152/528)	
Never	57.4% (128/223)	60.2% (318/528)	
Exposure to documented cases of COVID-19	19.6% (44/224)	18.2% (96/527)	0.68
Income deprivation score	0.102 (0.058 - 0.178)	0.105 (0.057 - 0.185)	0.98
Active disease (PRO2)	6.6% (14/212)	5.4% (27/497)	0.60
Time between vaccine doses (weeks)	11.0 (9.9 - 11.6)	11.0 (10.0 - 11.4)	0.72
Time from second dose to serum sample (weeks)	5.6 (3.9 - 7.6)	5.4 (3.6 - 7.7)	0.75

Supplementary Table 2: Baseline characteristics of participants who had a SARS-CoV-2 infection prior to receiving 2 doses of a SARS-CoV-2 vaccine

Abbreviations: anti-N = anti-nucleocapsid; anti-S RBD = anti-spike receptor binding domain; IBD = inflammatory bowel disease; 5-ASA = 5-aminosalicylic acid; BMI = Body Mass Index; PRO2 = IBD disease activity. Values presented are median (interquartile range) or percentage (numerator/denominator). All tests are two-sided. P values represent the results of a Mann Whitney U, Kruskal Wallis or Fisher’s exact test.

Variable	Infliximab	Vedolizumab	p
Vaccine			
BNT162b2	42.4% (329/776)	40.5% (314/776)	0.47
ChAdOx1 nCoV-19	57.6% (447/776)	59.5% (462/776)	
Age (years)	46.7 (34.5 - 59.2)	46.5 (34.6 - 59.9)	0.96
Sex			
Female	47.6% (371/779)	50.2% (389/778)	0.35
Male	52.4% (408/779)	49.9% (388/778)	
Prefer not to say	0.0% (0/777)	0.1% (1/778)	
Ethnicity			
White	91.0% (709/779)	90.4% (703/778)	0.93
Asian	5.8% (45/779)	5.8% (45/778)	
Mixed	1.9% (15/779)	2.4% (19/778)	
Black	0.6% (5/779)	0.5% (4/778)	
Other	0.6% (5/779)	0.9% (7/778)	
Diagnosis			
Crohn's disease	41.5% (323/779)	41.5% (323/778)	1.0
Ulcerative colitis	55.3% (431/779)	55.3% (430/778)	
IBD-unclassified	3.2% (25/779)	3.2% (25/778)	
Duration of IBD (years)	9.0 (4.0 - 17.0)	9.0 (4.0 - 16.0)	0.73
Age at IBD diagnosis (years)	31.6 (22.5 - 46.7)	32.1 (22.7 - 45.2)	0.72
Immunomodulators at recruitment	25.5% (199/779)	25.4% (198/778)	1.0
5-ASA at recruitment	30.9% (241/779)	32.5% (253/778)	0.51
Steroids in 2020	4.1% (32/779)	5.4% (42/778)	0.24
BMI	26.5 (23.3 – 30.0)	25.9 (23.1 - 29.7)	0.18
Heart disease	4.2% (33/779)	3.6% (28/778)	0.60
Diabetes	5.5% (43/779)	6.6% (51/778)	0.40
Lung disease	15.1% (118/779)	15.4% (120/778)	0.89
Kidney disease	1.0% (8/777)	1.4% (11/778)	0.50
Cancer	0.4% (3/779)	0.9% (7/778)	0.22
Smoker			
Yes	9.8% (76/779)	8.7% (68/778)	0.55
Not currently	33.9% (264/779)	36.2% (282/778)	
Never	56.4% (439/779)	55.0% (428/778)	
Exposure to documented cases of COVID-19	9.0% (70/779)	7.7% (60/778)	0.41
Income deprivation score	0.095 (0.054 - 0.154)	0.088 (0.053 - 0.146)	0.40
Active disease (PRO2)	10.0% (78/779)	9.1% (71/778)	0.61

Supplementary Table 3: Baseline characteristics of participants following propensity matching of baseline variables

Abbreviations: IBD = inflammatory bowel disease; 5-ASA = 5-aminosalicylic acid; BMI = Body Mass Index; PRO2 = IBD disease activity. Values presented are median (interquartile range) or percentage (numerator/denominator). All tests are two-sided. P values represent the results of a Mann Whitney U, Kruskal Wallis or Fisher's exact test.

Biologic and immunomodulator status	BNT162b2	ChAdOx1 nCoV-19
Infliximab with immunomodulator	94.1% (525/558)	93.1% (716/769)
Infliximab without immunomodulator	97.5% (347/356)	93.5% (557/596)
Vedolizumab with immunomodulator	98.7% (77/78)	99.3% (134/135)
Vedolizumab without immunomodulator	97.9% (328/335)	99.2% (479/483)

Supplementary Table 4: Proportion of patients with an anti-S RBD antibody concentration greater than the seroconversion threshold of 15U/mL at 2-10 weeks following two doses of SARS-CoV-2 vaccine stratified by biologic treatment, immunomodulator status and vaccine type

Values presented are percentage (numerator/denominator).

Type	Biologic: Infliximab	Vaccine: BNT162b2	Prior infection	Estimate (95% CI)	P value
Intercept				70.63 (69.37, 71.82)	<0.0001
Intercept	✓			-12.46 (-14, -10.99)	<0.0001
Intercept		✓		18.48 (16.59, 20.44)	<0.0001
Intercept			✓	6.72 (4.2, 9.24)	<0.0001
Intercept	✓	✓		-5.46 (-7.77, -3.15)	<0.0001
Intercept	✓		✓	-2.66 (-5.67, 0.35)	0.084
Intercept		✓	✓	-4.97 (-8.89, -1.12)	0.012
Intercept	✓	✓	✓	3.99 (-0.70, 8.68)	0.093
Gradient				-0.84 (-0.896, -0.784)	<0.0001
Gradient	✓			-0.53 (-0.595, -0.462)	<0.0001
Gradient		✓		-0.20 (-0.28, -0.119)	<0.0001
Gradient			✓	0.04 (-0.077, 0.168)	0.47
Gradient	✓	✓		-0.26 (-0.35, 0.161)	<0.0001
Gradient	✓		✓	0.11 (-0.035, 0.259)	0.15
Gradient		✓	✓	0.14 (-0.049, 0.322)	0.15
Gradient	✓	✓	✓	0.04 (-0.182, 0.259)	0.75

Supplementary Table 5: Summary statistics for each interaction term in the linear mixed model used to estimate anti-S RBD antibody half-life.

Modification of antibody half-life by biologic type, vaccine type and history of prior SARS-CoV-2 infection was evaluated by adding interaction terms in the model. All tests are two-sided. 95% confidence intervals of fixed effects and p values were calculated using likelihood ratios test.
