# Supplementary material for: Antibody decay, T cell immunity and breakthrough infections following two SARS-CoV-2 vaccine doses in inflammatory bowel disease patients treated with infliximab and vedolizumab
Source: Nat Commun. 2022 Mar 16;13:1379. doi: 10.1038/s41467-022-28517-z (PMC8927425; doi:10.1038/s41467-022-28517-z)
Supplement: Supplementary file 2 — Reporting Summary [file 41467_2022_28517_MOESM2_ESM.pdf]

nature portfolio

Corresponding author(s): Dr Tariq Ahmad

Last updated by author(s): Jan 7, 2022

Reporting Summary

Nature Portfolio wishes to improve the reproducibility of the work that we publish. This form provides structure for consistency and transparency in reporting. For further information on Nature Portfolio policies, see our Editorial Policies and the Editorial Policy Checklist.

Statistics

For all statistical analyses, confirm that the following items are present in the figure legend, table legend, main text, or Methods section.

n/a	Confirmed	
☐	☒	The exact sample size (*n*) for each experimental group/condition, given as a discrete number and unit of measurement
☐	☒	A statement on whether measurements were taken from distinct samples or whether the same sample was measured repeatedly
☐	☒	The statistical test(s) used AND whether they are one- or two-sided<br>*Only common tests should be described solely by name; describe more complex techniques in the Methods section.*
☐	☒	A description of all covariates tested
☐	☒	A description of any assumptions or corrections, such as tests of normality and adjustment for multiple comparisons
☐	☒	A full description of the statistical parameters including central tendency (e.g. means) or other basic estimates (e.g. regression coefficient) AND variation (e.g. standard deviation) or associated estimates of uncertainty (e.g. confidence intervals)
☐	☒	For null hypothesis testing, the test statistic (e.g. *F*, *t*, *r*) with confidence intervals, effect sizes, degrees of freedom and *P* value noted<br>*Give P values as exact values whenever suitable.*
☒	☐	For Bayesian analysis, information on the choice of priors and Markov chain Monte Carlo settings
☒	☐	For hierarchical and complex designs, identification of the appropriate level for tests and full reporting of outcomes
☐	☒	Estimates of effect sizes (e.g. Cohen's *d*, Pearson's *r*), indicating how they were calculated

Our web collection on statistics for biologists contains articles on many of the points above.

Software and code

Policy information about availability of computer code

Data collection	REDCap database (11.1.20) hosted at the Royal Devon and Exeter NHS Foundation Trust.
Data analysis	Statistical analyses were undertaken in R 4.1.2 (R Foundation for Statistical Computing, Vienna, Austria). Packages used include: rmarkdown (2.10), purr (0.3.4), ggplot2(3.3.5), dplyr (1.0.7), readr (2.0.1), forcats (0.5.1), tidyr(1.1.4), stringr(1.4.0), ggbeeswarm(0.6.0), forestmodel (0.6.2), cowplot(1.1.1), survminer(0.4.9), lme4(1.1-27.1), lmerTest(3.1-3), redcapAPI (2.3), MatchIt(4.3.2). Code has been made available at: https://github.com/exeteribd/clarityibd-public

For manuscripts utilizing custom algorithms or software that are central to the research but not yet described in published literature, software must be made available to editors and reviewers. We strongly encourage code deposition in a community repository (e.g. GitHub). See the Nature Portfolio guidelines for submitting code & software for further information.

Data

Policy information about availability of data

All manuscripts must include a data availability statement. This statement should provide the following information, where applicable:
- Accession codes, unique identifiers, or web links for publicly available datasets
- A description of any restrictions on data availability
- For clinical datasets or third party data, please ensure that the statement adheres to our policy

The study protocol including the statistical analysis plan is available at https://www.clarityibd.org/. Individual participant de-identified data that underlie the results reported in this article will be available immediately after publication for a period of 5 years. Due to the sensitive nature of the data, this will be made available to investigators whose proposed use of the data has been approved by an independent review committee. Analyses will be restricted to the aims in the approved proposal. Proposals should be directed to tariq.ahmad1@nhs.net. To gain access data requestors will need to sign a data access agreement. Data from the Virus

Watch study is currently being archived on the Office of National Statistics Secure Research Service and will be available shortly. Source data are provided with this paper.

Field-specific reporting

Please select the one below that is the best fit for your research. If you are not sure, read the appropriate sections before making your selection.

☒ Life sciences ☐ Behavioural & social sciences ☐ Ecological, evolutionary & environmental sciences

For a reference copy of the document with all sections, see nature.com/documents/nr-reporting-summary-flat.pdf

Life sciences study design

All studies must disclose on these points even when the disclosure is negative.

Sample size	The sample size for CLARITY IBD was based on the number of participants required to demonstrate a difference in the impact of infliximab and vedolizumab on seroprevalence and seroconversion following SARS-CoV-2 infection, with an estimated background seroprevalence of 0.05. We calculated that a sample of 6970 patients would provide 80% power to detect differences in the seroprevalence of SARS-CoV-2 antibodies in infliximab- compared with vedolizumab-treated patients, whilst controlling for immunomodulator status at the 0.05 significance level. Here, we report data from participants who had received uninterrupted biologic therapy since recruitment and had an antibody test performed between 14 and 70 days after a second dose of the either the BNT162b2 and ChAdOx1 nCoV-19 SARS-CoV-2 vaccines
Data exclusions	T cell data was excluded if response to the positive control anti-CD3 stimulation was <200 SFC per 10^6 PBMC.
Replication	Sensitivity analyses to control for the effect of immunomodulator use, and propensity matching to account for other significant differences in baseline variables were undertaken which replicated the primary findings. Rmarkdown was used to ensure reproducibility of all analyses.
Randomization	Not applicable - observational study.
Blinding	Not applicable - observational study

Reporting for specific materials, systems and methods

We require information from authors about some types of materials, experimental systems and methods used in many studies. Here, indicate whether each material, system or method listed is relevant to your study. If you are not sure if a list item applies to your research, read the appropriate section before selecting a response.

Materials & experimental systems

n/a	Involved in the study	
☒	☐	Antibodies
☒	☐	Eukaryotic cell lines
☒	☐	Palaeontology and archaeology
☒	☐	Animals and other organisms
☐	☒	Human research participants
☐	☒	Clinical data
☒	☐	Dual use research of concern

Methods

n/a	Involved in the study	
☒	☐	ChIP-seq
☒	☐	Flow cytometry
☒	☐	MRI-based neuroimaging

Human research participants

Policy information about studies involving human research participants

Population characteristics	Population characteristics are provided in Table 1
Recruitment	Consecutive patients were recruited at the time of attendance at infusion units from 92 National Health Service (NHS) hospitals across the UK between 22 September 2020 and 23 December 2020 . The eligibility criteria were age 5 years and over, a diagnosis of IBD, and current treatment with infliximab or vedolizumab for 6 weeks or more, with at least one dose of drug received in the previous 16 weeks. Patients were excluded if they had participated in a SARS-CoV-2 vaccine trial.
Ethics oversight	The sponsor was the Royal Devon and Exeter NHS Foundation Trust and the Surrey Borders Research Ethics committee approved the study (REC reference: REC 20/HRA/3114) in September 2020.

Note that full information on the approval of the study protocol must also be provided in the manuscript.

Clinical data

Policy information about clinical studies
All manuscripts should comply with the ICMJE guidelines for publication of clinical research and a completed CONSORT checklist must be included with all submissions.

Clinical trial registration	The study was registered with the ISRCTN registry (ISRCTN45176516) (https://doi.org/10.1186/ISRCTN45176516).
Study protocol	The protocol is available online at https://www.clarityibd.org.
Data collection	Consecutive patients were recruited at the time of attendance at infusion units from 92 National Health Service (NHS) hospitals across the UK between 22 September 2020 and 23 December 2020. Follow-up visits were timed to coincide with biological infusions and occurred approximately 8weekly. Variables recorded by participants were demographics (age, sex, ethnicity, comorbidities, height and weight, smoking status, and postcode), IBD disease activity (PRO2), SARS-CoV-2 symptoms aligned to the COVID-19 symptoms study (symptoms, previous testing and hospital admissions for COVID-19) and vaccine uptake (type and date of primary vaccination). Study sites completed data relating to IBD history (age at diagnosis, disease duration and phenotype according to the Montreal classifications, previous surgeries and duration of current biological and immunomodulator therapy). We linked our data by NHS number or Community Health Index to Public Health England, Scotland and Wales who archive dates and results of all SARS-CoV-2 PCR tests undertaken.
Outcomes	Our primary outcome was anti-S RBD antibodies 2 to 10 weeks after second dose of the BNT162b2 or ChAdOx1 nCoV-19 vaccines. Anti-S RBD antibody concentrations were reported as geometric means and standard deviations. Univariable analyses, using Spearman's rank correlation coefficients, and t-tests of log-transformed anti-S RBD antibody concentration were used to identify demographic, disease, vaccine, and treatment-related factors associated with the concentration of anti-S RBD antibodies across the cohort. Multivariable linear regression models were used to identify factors independently associated with log anti-S RBD concentration.<br><br>Secondary outcomes were:<br>(i)the proportion of participants who seroconverted<br>(ii) anti-spike T cell responses in patients following the first and second dose of vaccines<br>(iii) the durability of vaccine responses<br>(iv) risk of breakthrough infections two or more weeks after two doses of vaccine<br>(v) antibody concentrations and seroconversion rates in patients with PCR or serological evidence of past SARS-CoV-2 infection at, or prior, to the post-vaccination serum sample<br><br>Anti-spike T cell responses were assessed using Mann-Whitney U test to compare the magnitude of T cell response (SFC/10^6 PBMCs) stratified by treatment and vaccine received, and Spearman's rank correlation coefficient was calculated to determine correlation between antibody and T cell responses. Durability of vaccine responses were assessed using anti-S RBD antibody half-lives estimated using an exponential model of decay. Linear mixed models were fit using the lme4 and lmerTest package, with biologic treatment and vaccine type as fixed effects and each subject as a random effect. Each of these effects were estimated independently for gradient and intercept. 95% confidence intervals of fixed effects were calculated using likelihood ratios. P values for comparison of half-lives were estimated from the full linear mixed effects model that incorporated vaccine, biologic drug and prior SARS-CoV-2 infection status. Kaplan-Meier curves and cox proportional hazard regression model was used to identify treatment-related factors associated with time to breakthrough infection. Where appropriate the same analyses were used to compare antibody responses in participants with PCR evidence of SARS-CoV-2 infection at any time prior to vaccination.
